# FBXL10 contributes to the development of diffuse large B-cell lymphoma by epigenetically enhancing ERK1/2 signaling pathway

**DOI:** 10.1038/s41419-017-0066-8

**Published:** 2018-01-19

**Authors:** Xiujuan Zhao, Xing Wang, Qian Li, Wanbiao Chen, Na Zhang, Yu Kong, Junqiang Lv, Lei Cao, Dan Lin, Xi Wang, Guogang Xu, Xudong Wu

**Affiliations:** 1 Department of Cell Biology, 2011 Collaborative Innovation Center of Tianjin for Medical Epigenetics, Tianjin Key Laboratory of Medical Epigenetics, Tianjin Medical University, Tianjin, 300070 China; 2 Department of Bioinformatics, Tianjin Medical University, Tianjin, 300070 China; 3 Department of Immunology, Tianjin Medical University, Tianjin, 300070 China; 4 Nanlou Respiratory Department, PLA General Hospital, 28 Fuxing Road, Beijing, 100853 China; 5 Present Address: Department of Molecular Biology and Cell Biology, University of Science and Technology of China, Anhui, 230027 China

## Abstract

Epigenetic modifiers have emerged as critical factors governing the biology of different cancers. Herein we show that FBXL10 (also called KDM2B or JHDM1B), an important member of Polycomb repressive complexes, is overexpressed in human diffuse large B-cell lymphoma (DLBCL) tissues and the derived cell lines. Knocking down FBXL10 by specific short hairpin RNAs in DLBCL cells inhibits cell proliferation and induces apoptosis *in vitro*. Moreover, FBXL10 depletion in DLBCL cells abrogates tumor growth in mouse xenograft models. Through the analysis of RNA sequencing, we find that one of the key derepressed genes by depletion of FBXL10 is *DUSP6*, encoding a phosphatase for ERK1/2. Mechanistically FBXL10 maintains the silencing of *DUSP6* expression via recruitment of Polycomb group proteins and deposition of repressive histone modifications at the *DUSP6* promoter. Consistently, FBXL10 is required for ERK1/2 phosphorylation in DLBCL cells. Furthermore, we show that ERK1/2 activation and the proliferation rate of FBXL10-depleted cells can be rescued by downregulation of DUSP6 expression. These findings indicate that FBXL10 may be a promising therapeutic target in DLBCL and establish a link of epigenetic regulators to kinase signaling pathways.

## Introduction

Diffuse large B-cell lymphoma (DLBCL), the major prevalent B-cell non-Hodgkin lymphoma in adults worldwide, is characterized by heterogeneous genetic, phenotypic and clinical features. Despite of greatly improved outcomes over the past two decades^1^, a considerable proportion of DLBCL patients are still primarily refractory or experience short-term relapses impairing their possibilities of survival^2^. Understanding the potential molecular mechanisms are of great clinical importance for improved DLBCL treatment. Analogous to other malignancies, DLBCL harbors genomic lesions (deletions, amplifications and point mutations) that lead to oncogenic activation or to inactivation of tumor suppressor genes^3,4^. However, genetic lesions do not fully explain the molecular mechanisms underlying tumorigenesis and relapse of DLBCL. Growing research has suggested that epigenomic changes are a common hallmark of human cancers^5,6^. Accordingly, the aberrant expression or activity of chromatin modifiers is strongly linked to cancers. For example, Polycomb group (PcG) genes have been frequently found to be mutated or deregulated in malignancies^7,8^. Not surprisingly, epigenomic deregulations have been found to contribute to development of DLBCL^9,10^. Given the reversibility of epigenetic changes, improving our understanding of DLBCL by precise characterization of chromatin modifiers associated with the disease will be helpful to identify new therapeutic targets and develop novel strategies for effective treatments.

The majority of B cell lymphomas derive from germinal center (GC) B cells characterized by rapid proliferation and somatic hypermutation, DLBCL corresponds to B cells arrested by transformation events that occur at various stages of the GC transit^11^. On the basis of their gene expression profiles, the GC B cell (GCB)-like subtype of DLBCLs resemble light zone B cells, whereas activated B cell (ABC)-like DLBCLs seem to derive from GC cells arrested during the early stages of post-GC plasma cell differentiation^12^. Transcriptional repressor BCL6 plays its key role during the GC reaction by modulating a large number of pathways^13^, and the high expression of BCL6 mainly due to chromosomal translocations lead to the development of lymphomas^14^. BCOR is well known as one of the corepressors of BCL6^15^ and it forms a transcription repressive complex with PcG proteins^16,17^. Biochemistry studies have demonstrated that PcG proteins form at least two repressive complexes (PRC1 and PRC2). PRC1 and PRC2 are known to catalyze lysine 119 monoubiquitination of histone H2A (H2AK119ub1) and H3K27 tri-methylation (H3K27me3) respectively^18^, maintaining target genes in a silenced state^19^. Among the PRC1 complexes, we and others have confirmed that BCOR-PRC1 is the main E3 ligase complex responsible for H2AK119ub1^20,21^. Interestingly, this non-canonical PRC1 complex is necessary in GC B cells^22^.

FBXL10 (also called KDM2B or JHDM1B) is a member of non-canonical PRC1^17,21^, originally known as a demethylase against the dimethylation at lysine 36 of histone H3 (H3K36me2)^23^. Apart from a CxxC zinc finger that recognizes unmethylated CpG islands, it also contains a PHD domain, an F-box domain and a leucine-rich repeat (LRR) that participates in its incorporation into a non-canonical PRC1^16,21^. FBXL10 has been shown to play critical roles in tumorigenesis and self-renewal of cancer stem cells in solid tumors and hematopoietic malignancies^24–27^, but its role in lymphomagenesis is not clear by now.

In this study, we confirm that FBXL10 has oncogenic properties in DLBCL. Furthermore, we demonstrate that FBXL10-PRC1 maintains the silencing of BCL6 target genes such as *DUSP6* in DLBCL cells and therefore activates ERK1/2 to promote DLBCL cell proliferation. Thus, these findings provide insights into how the dysregulation of a chromatin modifier is coupled to sustaining proliferative kinase signaling pathway in DLBCL and also reveal the clinical importance of FBXL10 in DLBCL.

## Results

### FBXL10 expression is frequently upregulated in DLBCL cell lines and tumors

**Fig. 1:**
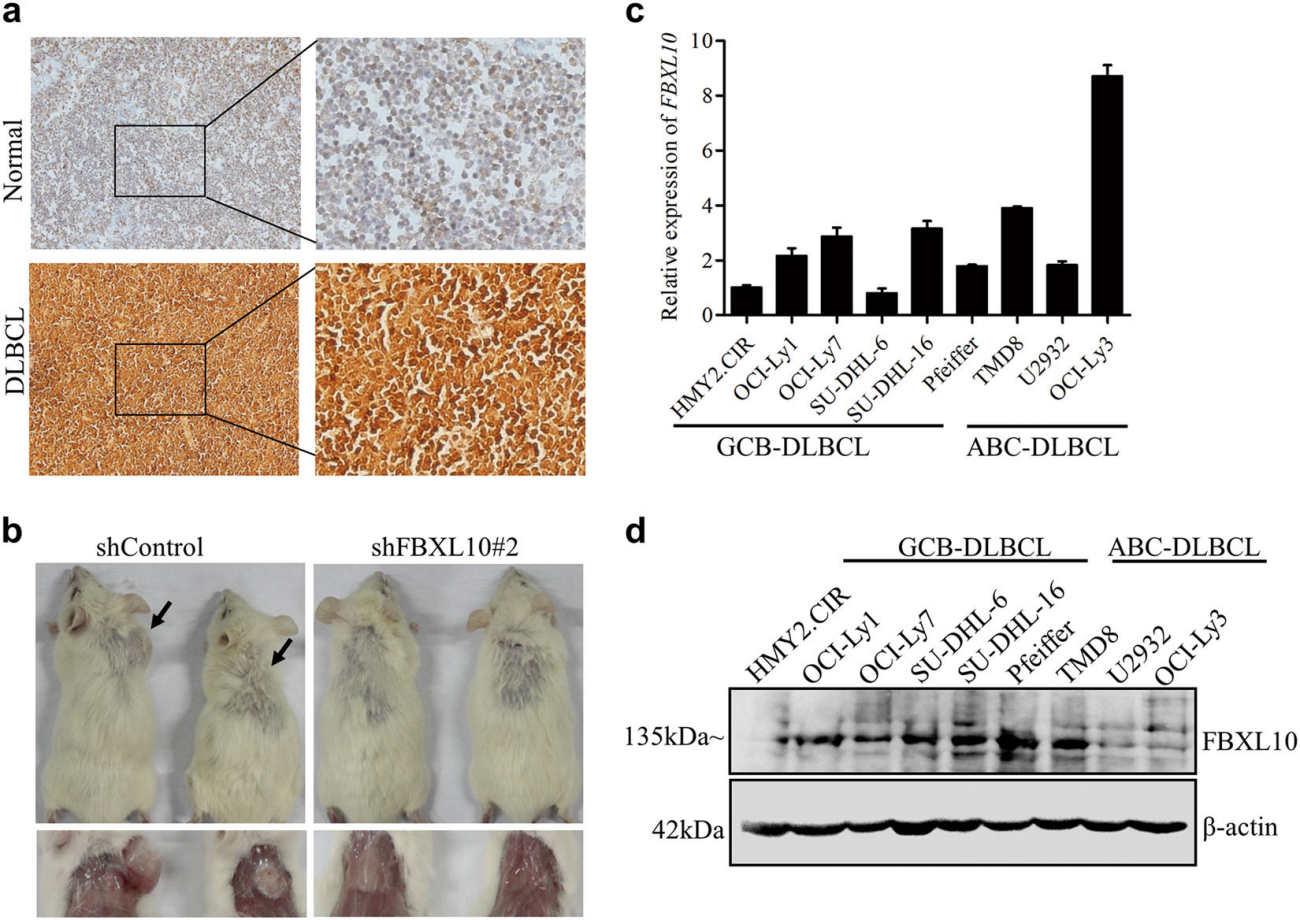
The expression pattern of FBXL10 in DLBCL tissue and cell lines **a** Representative photographs from IHC analysis of FBXL10 protein levels in normal and tumor samples (related to Table 1). **b** Representative pictures of tumors developed in NOD/SCID mice are shown, and tumors are indicated by black arrows. **c** qRT-PCR analysis of *FBXL10* mRNA in eight different DLBCL cells and one control cells. The error bars denote S.E.M., *n* = 3. **d** Western blots detection of FBXL10 protein in different DLBCL cells and control cells. β-actin served as a loading control for total protein

To investigate the relevance of FBXL10 expression to DLBCL development, we used immunohistochemical approach to detect FBXL10 expression on the tissue microarray composed of 124 surgical DLBCL samples and 16 normal lymph node samples. Based on the criteria defined in the Methods, the specimens were divided into two staining groups: low and high. As summarized in Table 1, FBXL10 levels are markedly elevated in more than half of all the DLBCL tumor samples compared with the normal lymph node controls (19%) (Fig. 1a). Similarly, high levels of FBXL10 expression were also observed in Burkitt lymphoma, follicular lymphoma (FL) samples (data not shown). We then examined FBXL10 expression in different established DLBCL cell lines. FBXL10 expression was generally elevated in most of the DLBCL cell lines at both mRNA (Fig. 1c) and protein levels (Fig. 1d), compared with the normal B cell line (HMy2.CIR). However, there is no difference of FBXL10 mRNA levels between GCB and ABC DLBCL no matter whether in the primary tumor tissues or relevant cells lines (Fig. 1c and Supplementary Fig. S1a).

**Table 1:** IHC analysis of FBXL10 protein levels in 124 DLBCL and 16 normal lymph node tissues

**IHC**	**FBXL10 expression**		
**Tissue**	**Low**	**High**	**Total**
**Normal**	13 (81%)	3 (19%)	16
**DLBCL**	58 (47%)	66 (53%)	124
**Total**	71	69	140

The levels of FBXL10 in DLBCL tumor tissues were classified as “low” or “high” as described in the Materials and Methods section

To evaluate the role of FBXL10 in tumorigenesis *in vivo*, we first treated OCI-Ly1 cells with shRNAs against FBXL10 using a lentiviral system and generated a stable FBXL10-depleted cell line. Then tumor growth abilities of control and FBXL10-depleted OCI-Ly1 cells was assessed using a mouse xenograft model on the basis of subcutaneous injection. The depletion of FBXL10 almost completely abrogated tumor growth (Fig. 1b and Supplementary Fig. S1b and c). These data confirm the pro-oncogenic roles of FBXL10 in DLBCL tumor progression.

### FBXL10 is critical for the survival and proliferation of DLBCL cells *invitro*

**Fig. 2:**
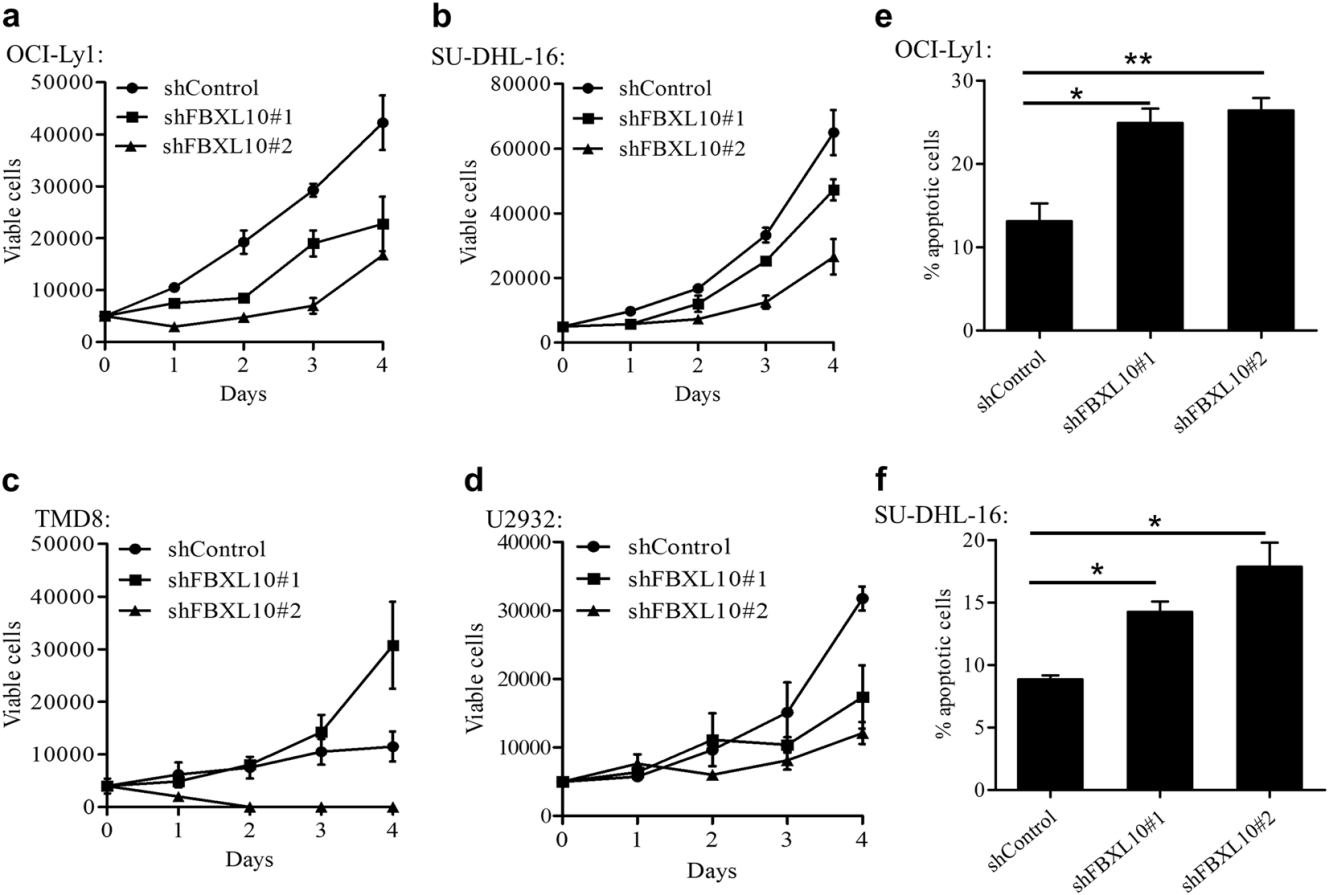
FBXL10 is critical for the survival and proliferation of DLBCL cells *in vitro* **a–d** Effect of FBXL10 knockdown on the proliferation of OCI-Ly1 (**a**), SU-DHL-16 (**b**), TMD8 (**c**) and U2932 (**d**) cells. Cells were transduced with shFBXL10#1 or #2 and shControl lentivirus. Cell proliferation was measured by trypan blue staining assays. **e–f** Representative apoptosis analysis using annexin-V/PI staining and flow cytometry of OCI-Ly1 (**e**) and SU-DHL-16 (**f**) cells transduced with shFBXL10#1 or #2 and shControl lentivirus for 48 h. The error bars denote S.E.M., *n* = 3. *P < 0.05 and **P < 0.01

We next sought to assess the potential roles of FBXL10 using DLBCL cell lines. First we tested 2 different shRNAs that efficiently knock down FBXL10 (Supplementary Fig. S2a) in selected DLBCL cell lines (OCI-Ly1, SU-DHL-16, TMD8 and U2932). The efficient depletion of FBXL10 led to consistent sharp reductions in anchorage-independent growth in GCB subtype of DLBCL cell lines (Fig. 2a, b). In contrast, variant effects between 2 shRNAs on the two of the ABC subtype of DLBCL cells were observed (Fig. 2c, d). These data indicate a requirement for FBXL10 especially in the proliferation of GCB DLBCL cells, exerts variable effects on ABC DLBCL cells. It has been previously reported NF-κB signaling directly upregulates FBXL10 transcription in human cancer cells^28^. Therefore the constitutive activation of the NF-κB pathway in ABC DLBCL cells probably bypasses the shFBXL10 effects in certain contexts.

Then we performed FACS analysis of cell cycles and cellular apoptosis in control and FBXL10-depleted GCB DLBCL cells. Although FBXL10 knockdown did not have any obvious effect on the cell cycles (Supplementary Fig. S2b, c), annexin-V/PI staining analysis demonstrated that suppression of FBXL10 significantly induced apoptosis in the GCB DLBCL cells (Fig. 2e, f). Taken together, these results suggest that FBXL10 is strictly required for the *in vitro* survival and growth of GCB DLBCL cells.

### FBXL10 regulates transcriptional programs involved in cell identity and cell metabolism

**Fig. 3:**
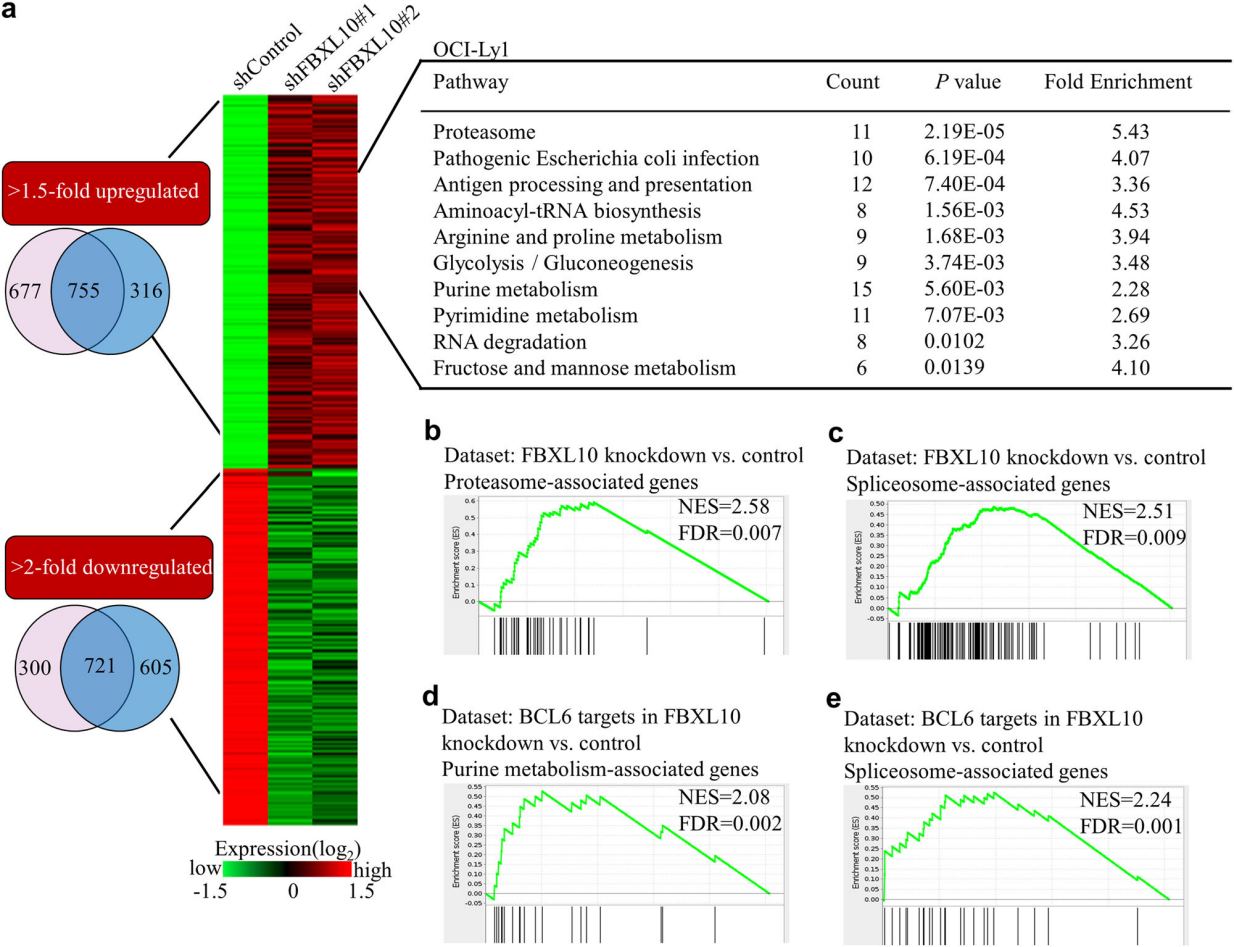
RNA-seq analysis of FBXL10 deficiency in DLBCL cells **a** Venn diagrams and a heat map of genes that are consistently 1.5-fold upregulated (top) or 2-fold downregulated (bottom) by FBXL10 knockdown. Pathway analysis of upregulated genes is shown on the right. **b** GSEA of proteasome genes from human FBXL10 knockdown OCI-Ly1 cells compared with control cells. The normalized enrichment score (NES) reflects the degree to which a gene set is upregulated (positive NES) or downregulated (negative NES). Corresponding *P* values are indicated. **c** GSEA of genes associated with spliceosome from human FBXL10 knockdown OCI-Ly1 cells compared with control cells. **d-e** GSEA of BCL6-specific regulated genes in expression data from FBXL10-deficient OCI-Ly1 cell compared with control cells

To get insight into how FBXL10 knockdown affects cell growth, RNA sequencing was performed on the OCI-Ly1 shFBXL10#1, shFBXL10#2 cells and control cells. Bioinformatics analysis revealed 1476 differentially expressed genes (*P* < 0.05, fold change >1.5 for upregulated genes and fold change >2.0 for downregulated genes); with 755 genes upregulated and 721 genes downregulated upon FBXL10 knockdown (Fig. 3a and Supplementary Table S3). Two-way hierarchical cluster analysis revealed that FBXL10 knockdown cells displayed different gene expression patterns from control cells (Fig. 3a). Functional analysis of 755 upregulated genes revealed that FBXL10 downregulation mainly affected pathways for immune regulation and protein and RNA processing (Fig. 3a). Similarly, Gene set enrichment analysis (GSEA) supported the established role of FBXL10 as a regulator of genes whose functions were related with protein degradation (Fig. 3b) and RNA splicing (Fig. 3c). Hence in human DLBCL cells, FBXL10 controls a common set of genes related to cell metabolism and protein degradation pathways.

It has been demonstrated that the BCL6 transcriptional repressor is required for development of GC B cells and the constitutive expression causes diffuse large B-cell lymphomas^29^. Moreover, BCL6 and the BCOR-PRC1 complex containing FBXL10 are necessary for EZH2-driven GC formation and lymphoma precursor lesions^22^. Therefore we would like to find out whether there exist functional interactions between FBXL10 and BCL6 in DLBCL. Statistical analysis based on TCGA database indicated a positive correlation between the expression of FBXL10 and BCL6 (Supplementary Fig. S3a). We also validated the upregulated BCL6 expression in different DLBCL cancer cells (Supplementary Fig. S3b, c) and confirmed that FBXL10 interacted with BCL6 (Supplementary Fig. S3d).

Based on the study that 1564 genes are specifically bound by BCL6 in DLBCL cells compared with GC B cells^30^, we next analyzed the BCL6 targets in FBXL10 knockdown OCI-Ly1 cells and control cells. Interestingly, we found this subset of genes were similarly enriched in spliceosome and metabolism pathway (Fig. 3d, e), which supports that FBXL10 and BCL6 cooperate to regulate downstream target genes in DLBCL cells.

### The BCL6 target genes such as *DUSP6* is silenced by FBXL10 in human DLBCL

**Fig. 4:**
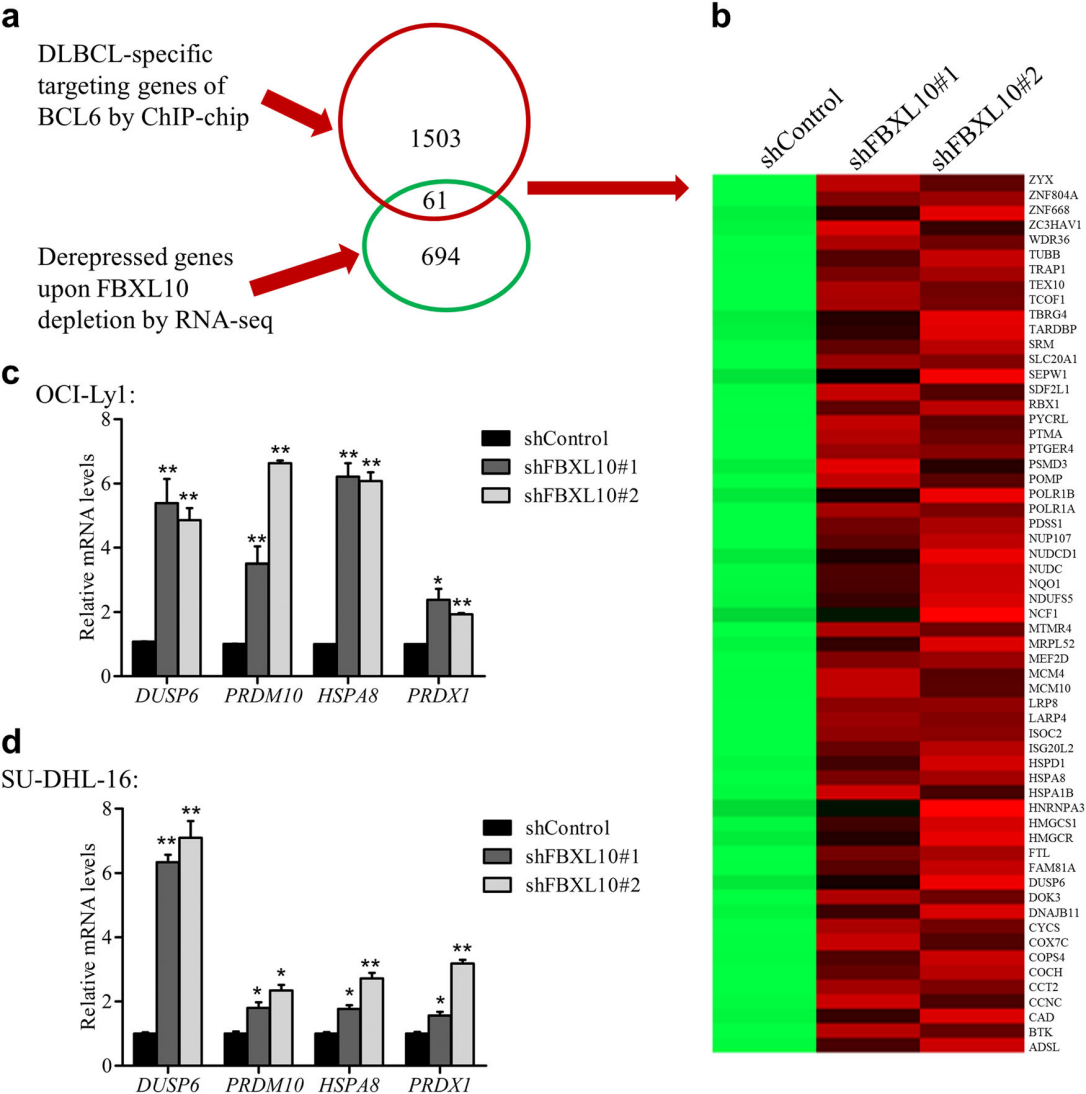
The BCL6 target genes such as *DUSP6* is derepressed by FBXL10 downregulation in DLBCL cells **a** Workflow analysis to identify potential cotarget genes for BCL6 and FBXL10 in DLBCL. **b** A heatmap of genes whose promoters containing BCL6-binding sites and regulated by FBXL10 meanwhile. See also Supplementary Table S3**. c–d** Expression levels of FBXL10 target genes (*DUSP6, HSPA8, PRDM10*, and *PRDX1*) in OCI-Ly1 (**c**) and SU-DHL-16 (**d**) cells after FBXL10 knockdown. The mRNA levels were analyzed by quantitative RT-PCR. The error bars denote S.E.M., *n* = 3. *P < 0.05, and **P < 0.01

To further investigate the specific targets and underlying molecular mechanisms of FBXL10 in DLBCL, we would like to identify the potential candidate genes commonly targeted by FBXL10 and BCL6 in DLBCL. Notably, 61 genes among the 755 upregulated genes (Fig. 4a, green circle) were overlapped with BCL6 target genes in DLBCL (Fig. 4a, red circle). These 61 potential FBXL10 and BCL6 cotarget genes were mainly associated with protein and nucleoside binding functionally (Fig. 4b). Among the regulated genes, the derepression *DUSP6*, *PRDM10*, *HSPA8*, and *PRDX1* were individually confirmed by quantitative RT-PCR assays in the GCB DLBCL cells (Fig. 4c, d), while inconsistent derepression were observed in the ABC DLBCL cells (Supplementary Fig. S4a, b). Of such genes, *DUSP6* expression was notably (more than 5-fold) upregulated by FBXL10 depletion in the two GCB DLBCL cells but not in the ABC DLBCL cells. This is also supported by the previous reports of DUSP6 with tumor suppressive roles^31,32^. Taken together, we identify *DUSP6* as one of the potential key FBXL10 target genes.

### FBXL10 regulates histone modifications at the *DUSP6* promoter via recruitment of PcG proteins

**Fig. 5:**
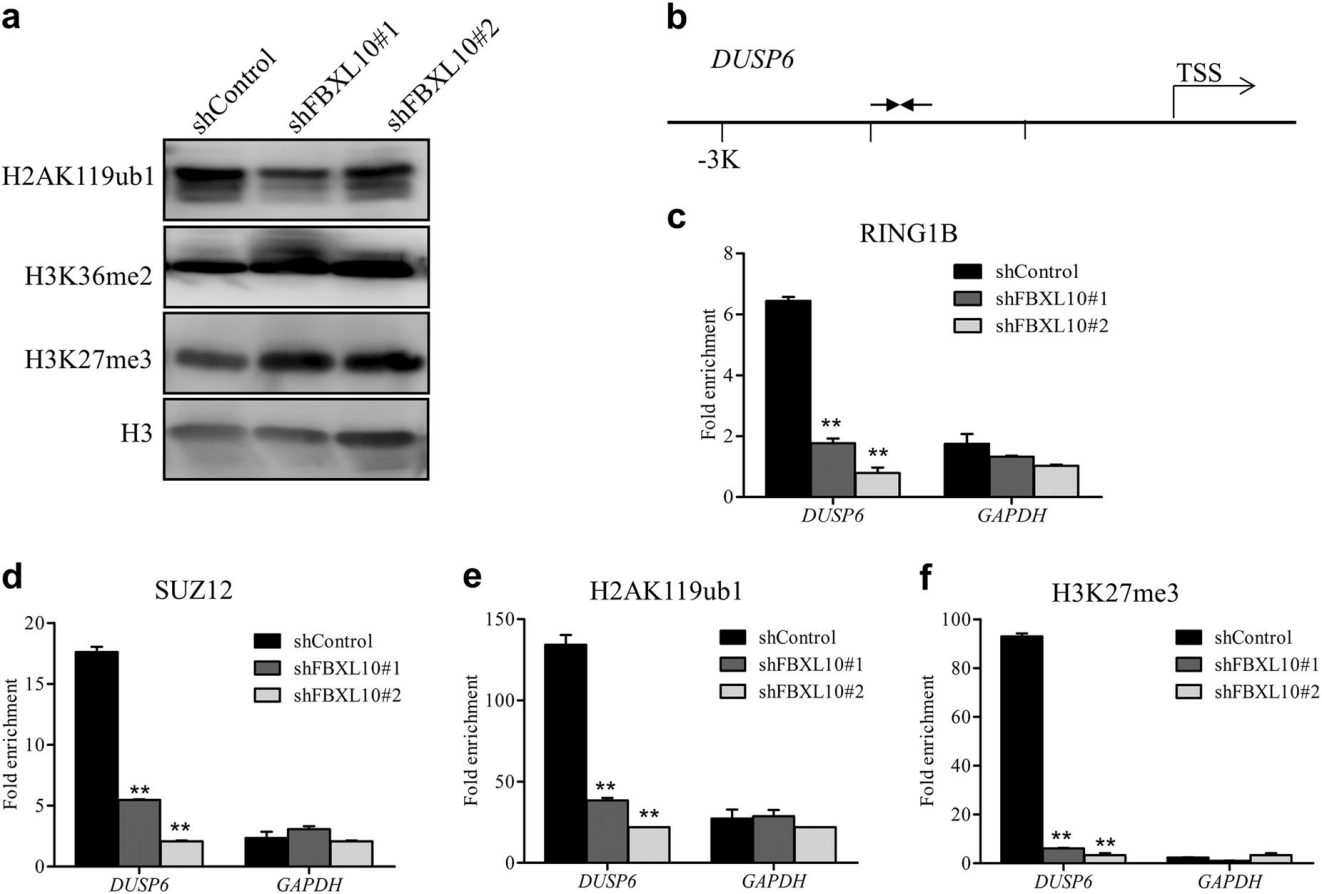
FBXL10 is required for PcG protein recruitment and deposition of repressive histone modifications to *DUSP6* promoter **a** Western blot analysis of designated histone modification levels in OCI-Ly1 cells transduced with shControl and shFBXL10#1 or #2 virus. H3 served as a loading control for total protein. **b** Diagrammatic representation of the promoter regions of the *DUSP6* gene. Arrows indicated the PCR-amplified regions. TSS, transcription start site. **c–f** ChIP-qPCR analyses of RING1B (**c**), SUZ12 (**d**), H2AK119ub1 (**e**), H3K27me3 (**f**) occupancy on the CpG island of *DUSP6* promoter in shControl and FBXL10-depleted DLBCL cells. The *GAPDH* promoter was used as negative controls. The error bars denote S.E.M., *n* = 3, **P < 0.01

Next we examined the levels of histone modifications in DLBCL cells after downregulating FBXL10 expression. We found that H2AK119ub1 level was significantly reduced in FBXL10 depleted cells compared with control cells, but the levels of H3K36me2 and H3K27me3 remained unchanged (Fig. 5a). Furthermore, we performed ChIP-qPCR analysis to investigate the potential epigenetic mechanisms for the regulation of DUSP6 by focusing on the promoter of *DUSP6* (Fig. 5b). In control DLBCL cells, the core members of PcG (RING1B and SUZ) were strongly enriched at the promoter of *DUSP6*. In contrast, their binding was disrupted upon depletion of FBXL10 (Fig. 5c, d). Accordingly the levels of H2AK119ub1 and H3K27me3 were significantly decreased (Fig. 5e, f) in FBXL10-depleted cells. Taken together, these results demonstrate that FBXL10 is responsible for recruitment of PcG proteins and deposition of repressive histone modifications to the promoter of *DUSP6* and therefore the maintenance of transcriptional repression.

### Transcriptional repression of *DUSP6* by FBXL10 contributes to the activation of ERK1/2 signaling pathway and DLBCL cell proliferation

**Fig. 6:**
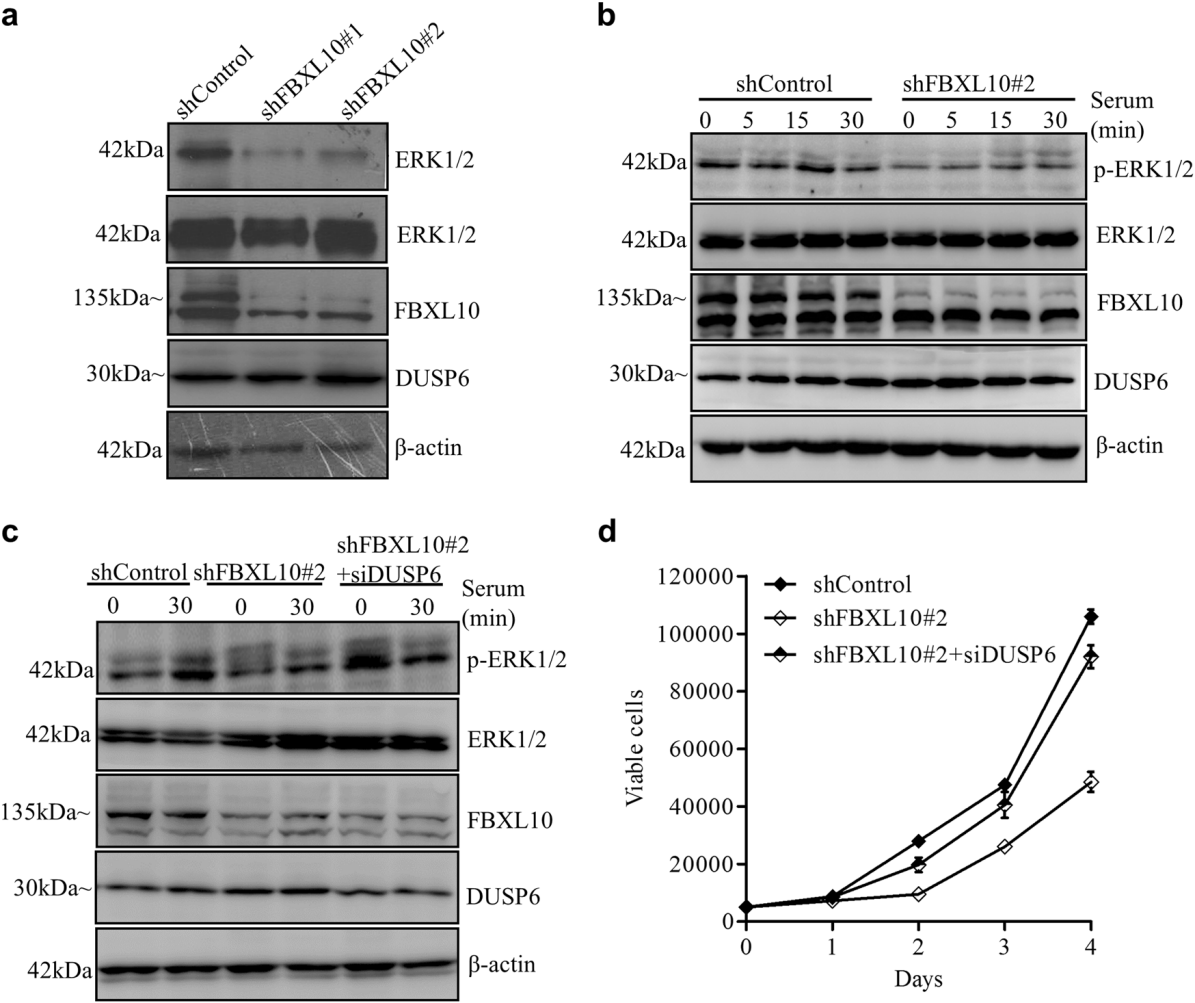
The transcriptional repression of *DUSP6* by FBXL10 leads to ERK1/2 activation and facilitates the proliferation of DLBCL cells **a** Effect of FBXL10 knockdown on phosphorylation level of ERK1/2. OCI-Ly1 cells transduced with shControl or shFBXL10#1 or #2 virus were examined by Western blot analysis with indicated antibodies. **b** Effect of FBXL10 knockdown on phospho-ERK1/2 levels during serum activation. FBXL10 depleted OCI-Ly1 cells and control cells were stimulated with 10% serum for 5, 15, or 30 min after 18 h of serum starvation. Protein extracts were then examined by Western blot analysis. **c** Effect of double knockdown of FBXL10 and DUSP6 on phosphorylation levels of ERK1/2 during serum activation. Control, FBXL10-depleted, and DUSP6/FBXL10-depleted OCI-Ly1 cells were stimulated with 10% serum for 30 min. For **a**–**c**, β-actin served as a loading control for total protein. **d** Rescue of the proliferation defect of FBXL10-depleted OCI-Ly1 cells by DUSP6 knockdown. The cell numbers were determined at 1–4 days using trypan blue staining for the indicated groups. The error bars denote S.E.M., *n* = 3

DUSP6 has been shown to specifically prevent the activation of ERK1/2 by dephosphorylation of both the threonine and tyrosine residues in mouse embryonic fibroblasts^33^. ERK1/2 is a member of Mitogen-activated protein kinases (MAPKs) that play a critical role in mediating cellular signaling events, including cell proliferation and invasion^34^. Moreover, ERK1/2 has been described constitutively active and playing important role in the oncogenesis of B cell tumors^35^. We next assessed whether the derepression of *DUSP6* due to FBXL10 depletion confers negative effects on the phosphorylation of ERK1/2. Western blot analysis showed that FBXL10 knockdown resulted in dramatic decrease of phospho-ERK1/2 levels though DUSP6 protein levels were only slightly increased (Fig. 6a). To further see the relationship in a dynamic fashion, the control or FBXL10-depleted OCI-Ly1 cells were first cultured with serum free medium. After reintroducing serum, we could see a rapid gain of ERK1/2 phosphorylation in the control cells. However the depletion of FBXL10 strongly delayed the phosphorylation of ERK1/2 (Fig. 6b). These results indicate that FBXL10 is required for the activation of ERK signaling pathway by shutting off *DUSP6* expression in DLBCL cells.

To further determine whether ERK1/2 inactivation upon FBXL10 downregulation is dependent on the derepression of DUSP6, we depleted DUSP6 in FBXL10 knockdown OCI-Ly1 cells by siRNA, and then starved these double-knockdown cells (shFBXL10#2+siDUSP6) in addition to shControl cells and shFBXL10#2 cells. After reintroducing serum into the culture medium, we compared phospho-ERK1/2 levels among these 3 groups of OCI-Ly1 cells during serum activation. Analysis of phospho-ERK1/2 levels showed that DUSP6 knockdown restored phospho-ERK1/2 levels in FBXL10-depleted cells (Fig. 6c). Next, we examined the effect of DUPS6 knockdown on the proliferation of FBXL10-depleted cells. Interestingly, DUSP6 knockdown significantly restored the defective proliferation of FBXL10 depleted cells (Fig. 6d). These data suggest that the proliferation of FBXL10-overexpressing DLBCL cells is largely dependent on the transcriptional repression of *DUSP6* by FBXL10.

## Discussion

**Fig. 7:**
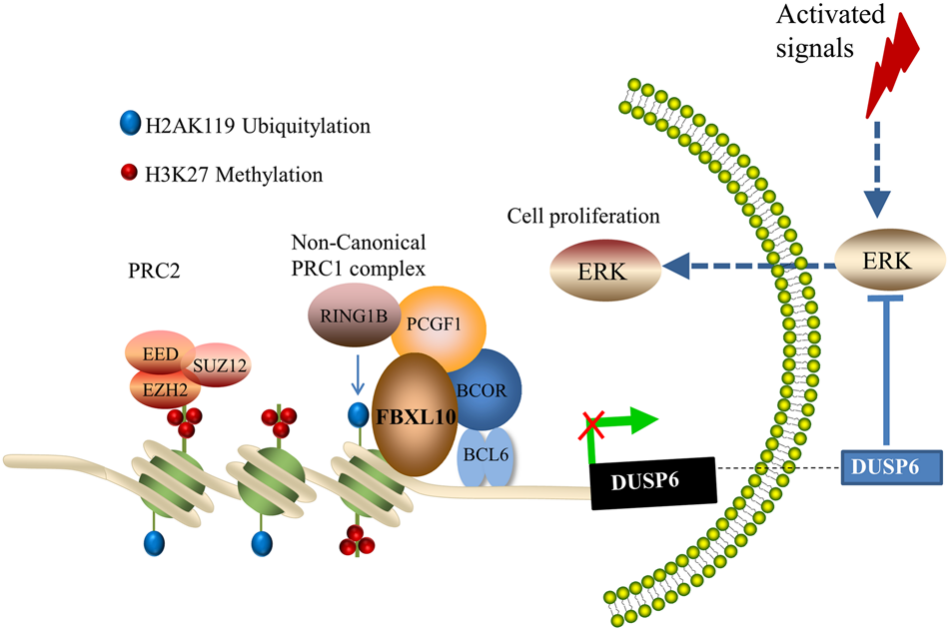
A hypothetical representation of the regulatory mechanism underlying FBXL10-promoted DLBCL cell proliferation The phosphatase DUSP6 specifically dephosphorylates ERK1/2. FBXL10 represses the BCL6 target gene *DUSP6* by recruiting PcG proteins and deposition of repressive histone modifications such as H2AK119ub1 and H3K27me3. The transcriptional repression of *DUSP6* hence results in increased phospho-ERK1/2 levels, which are critical for cell proliferation in DLBCL cells

The contribution of epigenomic alterations to tumor progression and relapse has gradually been well recognized by now. To improve the therapeutic efficacy of DLBCL, it will be meaningful to identify critical chromatin modifiers and understand their tumorigenic mechanisms. In this study, we unveil that FBXL10 is a pivotal driver of an epigenetic program that is critical for the tumorigenicity of DLBCL. We provide evidences that FBXL10 accomplishes oncogenic functions through the coordinated repression of BCL6 target genes such as *DUSP6* in DLBCL. FBXL10 and PcG proteins form a transcription repressive complex, which recognizes and binds to the promoter region of *DUSP6* and maintains the silenced state by catalyzing H2AK119 monoubiquitination and H3K27 trimethylation. Thus, activation of ERK1/2 by FBXL10-mediated repression of *DUSP6* contributes to the proliferation and survival of DLBCL cells (Model shown in Fig. 7).

The aberrant activation of sustaining growth signaling is one of the hallmarks of cancers. The MAPK/ERK pathway participates in the generation of mitogenic responses in essentially all hematologic malignancies^36^. However, the roles of chromatin modifiers in regulating the oncogenic signaling pathway are barely investigated. Interestingly, this study defines a critical role for FBXL10 in DLBCL pathogenesis by activating a pro-survival ERK signaling pathway via transcriptional repression of *DUSP6*. Of note, DUSP6 is frequently inactivated in solid tumors and is specifically required for the dephosphorylation of ERK1/2^33,37^. Consistent with this, we show that DUSP6 knockdown in FBXL10-depleted cells rescues phospho-ERK1/2 levels during serum activation and restores the growth of FBXL10-depleted cells. In addition, FBXL10 downregulation may not lead to cellular apoptosis via caspase 3 activation, because G1 cell population and caspase 3 cleavage are not modulated by FBXL10 knockdown (data not shown). Thus, the activation ERK1/2 signaling pathway is one of the main pro-oncogenic mechanisms for FBXL10 in DLBCL. Our data also support therapeutic use of the ERK inhibitors as a promising approach in DLBCL^35,38^.

We and others have previously shown that FBXL10 promotes H2AK119 monoubiquitylation via recruiting PcG proteins to the promoters of target genes^21,39,40^. Here we demonstrate that downregulation of FBXL10 results in a decrease of PcG binding and the associated H2AK119ub1 and H3K27me3 levels at the *DUSP6* promoter, thus confirming *DUSP6* as a direct target of FBXL10 in DLBCL cells. Notably, the enrichment level of H3K36me2 is almost unchanged by FBXL10 loss, possibly due to the compensation of FBXL11, a close homolog of FBXL10^41^. As *DUSP6* is one of BCL6 target genes in DLBCL cells, it is likely that FBXL10-mediated BCOR-PRC1 binding is coupled with BCL6, a DNA sequence-specific transcriptional suppressor^30^. In addition, FBXL10 may play a broader role in the progression of lymphoma because our RNA-seq analyses have unveiled many other upregulated genes such as the genes related to amino acids metabolism after the depletion of FBXL10. As signaling pathways implicated in cell proliferation usually regulate metabolic states in cancer cells^42^, the deregulated DUSP6-ERK axis may underlie the aberrant cell metabolisms. Further work will be carried out to explore these potential links in the future.

Two main subtypes of DLBCL show distinct genetic aberrations, raising the notion that distinct mechanisms may regulate their survival^11^. Notably here we find that FBXL10 is critical to the survival of GCB DLBCL not ABC DLBCL cells. Apparently this is not due to the expression differences in the two subtypes (Supplementary Fig. S1a). The functional differences are more likely due to the distinct origin of development stages. The non-canonical PRC1-BCOR complex including FBXL10 has been known to play a dominant role in GC B cells that GCB DLBCL presumably derives from^11,22^. On the other hand, we and others have found EZH2 inhibitors only were against to GCB not useful for ABC DLBCL cells*in* *vivo* and *in* *vitro*^43^. Therefore it seems that FBXL10 and the Polycomb-dependent silencing mechanism play a more important role in the pathogenesis of GCB DLBCL than the ABC subtype. Indeed, FBXL10 is not only highly expressed in 53% of DLBCL patient samples examined (Table 1), but also overexpressed in FL and Burkitt lymphoma that both originate GC B cells whether in patient samples or cell lines (data not shown). That means it may be a general mechanism to promote survival B cell lymphomagenesis derived from GC B cells, not just exclusively be restricted to DLBCL transformed from FL.

In summary, our study has identified a key epigenetic player in the pathogenesis of DLBCL by a different mechanism. Down-regulation of FBXL10 also results in significant degree of antitumor activity in mouse models of disease. Therefore our findings provide a rationale for the development of novel small inhibitors directed toward FBXL10 in FBXL10-overexpressing lymphoma.

## Materials and methods

### Culture of DLBCL cell lines

The normal B cell line HMy2.CIR was a kind gift from Dr. Xiaoyue Tan^44^. OCI-Ly7 and OCI-Ly3 cells were cultured in IMDM media with 10% fetal bovine serum and 1% penicillin–streptomycin (Gibco, Grand Island, NY, USA), and the other cells (OCI-Ly1, SU-DHL-6, SU-DHL-16, Pfeiffer, TMD8, and U2932) were cultured in RPMI-1640 media with 10% serum and 1% penicillin–streptomycin. Cell lines were authenticated by examination of morphology and growth characteristics and confirmed to be mycoplasma free.

### shRNAs and siRNA

To generate stable FBXL10 knockdown cells, OCI-Ly1 cells were infected with shFBXL10-containing viruses. Sequences for shFBXL10#1 and shFBXL10#2: GAAGGCAAGTTTAACCTCATG, CTGAACCACTGCAAGTCTATC. Scramble- containing virus infected cells were used as control. Cells were selected in 2 μg/ml puromycin containing medium. For the rescue assay, siRNAs against *DUSP6* and scramble siRNA were purchased from Sigma-Aldrich. shFBXL10#2-treated cells (1 × 10^6^) in a 6-well plate were nucleofected with the siRNAs using the SE Cell Line 4D-Nucleofector™ X Kit (Lonza, Basel, Switzerland,) according to the manufacturer’s protocol. Then cells were harvested for protein analysis and cell proliferation assay after 24–48 h of incubation.

### RNA isolation and quantitative RT-PCR

Total RNA was isolated using Trizol reagent (Invitrogen, Carlsbad, USA). Total RNA (1 μg) was used for synthesis of first-strand cDNA using M-MLV reverse transcriptase (ThermoFisher, Beijing, China). Quantitative real-time PCR was performed using the SYBR green mix (Roche, Basel, Switzerland). The reactions were performed in triplicates with Applied Biosystems 7500 Fast Real-Time PCR System. Data were displayed as 2^- ΔΔCT^ values. Specific primers for FBXL10, β-actin (control) and other genes were listed in Supplementary Table S1.

### Antibodies and immunoprecipitation analysis

The FBXL10-specific antibodies were generated by immunizing rabbits with GST-FBXL10 (amino acids 400–600 and 717–964 of human FBXL10), and were subsequently affinity purified. Antibody specificity was confirmed by immunoblot, immunoprecipitation and immunohistochemistry. Total cell lysates (40 µg) were subjected to 10% SDS-PAGE gel electrophoresis and were blotted with series of antibodies. Antibodies for HA, H3K36me2, H3K27me3, H2AK119ub1, ERK1/2, phospho-ERK1/2 (Thr202/Tyr204) were listed in Supplementary Table S2. For immunoprecipitations, cells were lysed in lysis buffer (50 mM Tris-HCl at pH 7.4, 0.2 mM EDTA, 150 mM NaCl, 3% NP-40, 1 mM phenylmethylsulfonyl fluoride (PMSF) and protease inhibitor cocktail). Equal amounts of cell extracts were incubated with anti-FBXL10 antibodies followed by subsequent immunoblots.

### Immunohistochemical staining

Formalin-fixed and paraffin embedded tissue microarray samples were obtained from Xian Alena Biotech Co., Ltd. Paraffin sections were incubated at 4 °C overnight with the FBXL10 antibody after dewaxing and hydration. Slides were incubated with a biotinylated secondary antibody for 1.5 h, then developed with avidin-peroxidase and DAB and counterstained with hematoxylin. Dehydrated slides were mounted with neutral resin. All slides were evaluated independently by two investigators without any prior knowledge of each patient’s clinical information. The staining results of FBXL10 were scored using the following criteria: (i) percentage of positive tumor cells in the tumor tissue: 0 (<5%), 1 (5%–25%), 2 (26%–50%), 3 (51%–75%) and 4 (>75%); and (ii) staining intensity: zero (no signal), 1 (weak), 2 (moderate) and 3 (strong). Final scores were calculated by multiplying the score for the percentage of positive cells by the intensity score (range 0–12). The levels of FBXL10 protein in each sample were further classified as low (final score < median level) or high (final score ≥ median level).

### Analysis of proliferation and apoptosis

Cells were seeded at a density of 5×10^3^ cells per well in 96-well plates. Relative cell numbers were determined at 1–4 days using trypan blue staining. For annexin-V/ propidium iodide (PI) analysis, cells were stained according to the manufacturer’s instructions (BD Biosciences, San Jose, CA) and positive cells for annexin-V detection were measured using FlowJo software (BD Biosciences, San Jose, CA).

### Gene-expression analysis

For RNA sequencing (RNA-seq), two groups of stable FBXL10 knockdown cells and control cells were harvested and RNAs were extracted using Trizol reagent followed by a genomic DNA elimination step. RNA size, concentration and integrity were verified using Agilent 2100 Bioanalyzer (Agilent Technologies). Library construction and sequencing on BGISEQ-500 was performed at Beijing Genomics Institute (BGI). All the generated raw sequencing reads were filtered to remove reads with adapters, reads in which unknown bases are more than 10%, and low quality reads. Clean reads were then obtained and stored as FASTQ format. HISAT^45^ was used to map clean reads to the genome of hg19. Gene expression levels are quantified by a software package called RSEM^46^. NOISeq method^47^ was used to screen differentially expressed genes between two groups.

### Quantitative chromatin immunoprecipitation (ChIP) assay

DLBCL Cells were chemically cross-linked with 1% formaldehyde solution for 10 min at room temperature with gentle agitation and quenched with 0.125 M glycine. Then the fixed cells were resuspended, lysed, and sonicated to solubilize and shear crosslinked DNA. Then samples were ultra-sonicated for 25 min with 30 s ultra-sonication at 30 s intervals (Bioruptor pico, Diagenode, Belgium). The resulting fragmented chromatin extract was precleared with Protein A/G beads (ThermoFisher, Beijing, China) and then incubated overnight with H2AK119ub1, RING1B, H3K27me3 and SUZ12 antibodies separately followed by washes, elution and reverse cross-linking. DNA was purified using PCR purification kits (QIAGEN, Hilden, Germany), and amplified by quantitative PCR using specific primer sets for individual genes (see Supplementary Table S1). Ct values were normalized to input and calculated as percentage of input. Relative occupancy indicated the fold change in percentage of input over the control.

### Mouse xenograft studies

Male NOD/SCID mice aged 6–8 weeks were purchased from the HFK Bioscience CO., LED (Peking, China). All animal experiments were performed according to Health guidelines of Tianjin Medical University Institutional Animal Use and Care Committee. Mice were subcutaneously injected with low-passage 1×10^7^ human OCI-Ly1 stable knockdown and control cells. Tumor volume was monitored every other day with electronic digital calipers (Fisher Scientific) in two dimensions. Tumor volume was calculated with the formula: tumor volume (mm^3^) = (smallest diameter^2^×largest diameter)/2^29^. All mice were sacrificed and tumors tissues were harvested and weighed when one of each pair reached the maximal tumor mass permitted by our protocol.

### Statistical analysis

Data were analyzed by Student's *t*-test when comparing two groups. Data were plotted as mean ± S.E.M (standard error of the mean) and *P*-values were calculated. * and ** denote *P* < 0.05 and *P* < 0.01 in the figures.

## Electronic supplementary material

Figure Legends and Tables for Supplementary Material

Figure S1

Figure S2

Figure S3

Figure S4
