# Supplementary material for: FBXL10 contributes to the development of diffuse large B-cell lymphoma by epigenetically enhancing ERK1/2 signaling pathway
Source: Cell Death Dis. 2018 Jan 19;9(2):46. doi: 10.1038/s41419-017-0066-8 (PMC5833345; doi:10.1038/s41419-017-0066-8)
Supplement: Supplementary file 1 — Figure Legends and Tables for Supplementary Material [file 41419_2017_66_MOESM1_ESM.doc]

**FBXL10 contributes to the development of diffuse large B-cell lymphoma by epigenetically enhancing ERK1/2 signaling pathway**

Xiujuan Zhao^1^, Xing Wang^1^, Qian Li^1^, Wanbiao Chen^2, #^, Na Zhang^2^, Yu Kong^1^, Junqiang Lv^3^, Lei Cao^1^, Dan Lin^2^, Xi Wang^1^, Guogang Xu*^,4^ , Xudong Wu*^,1^

^1^Department of Cell Biology, 2011 Collaborative Innovation Center of Tianjin for Medical Epigenetics, Tianjin Key Laboratory of Medical Epigenetics, Tianjin Medical University, Tianjin 300070, China

^2^Department of Bioinformatics, Tianjin Medical University, Tianjin 300070, China

^3^Department of Immunology, Tianjin Medical University, Tianjin 300070, China

^4^Nanlou Respiratory Department, PLA General Hospital, 28 Fuxing Road, Beijing100853, China

### ^#^ Present address: Department of Molecular Biology and Cell Biology, University of Science and Technology of China, Anhui 230027, China

*Correspondence author: Guogang Xu, Nanlou Respiratory Department, PLA General Hospital, 28 Fuxing Road, Beijing100853, China. Tel and Fax: +86 10 6687 6250; Email address: [xuguogang@gmail.com](mailto:xuguogang@gmail.com); Xudong Wu, Department of Cell Biology, 2011 Collaborative Innovation Center of Tianjin for Medical Epigenetics, Tianjin Key Laboratory of Medical Epigenetics, Tianjin Medical University, Tianjin 300070, China. Tel: +86 22 8333 6825; Fax: +86 22 8333 6519; Email address: [wuxudong@tmu.edu.cn](mailto:wuxudong@tmu.edu.cn)

**Supplementary Figure Legends**

**Figure S1** **Depletion of FBXL10 abrogates tumorigenicity of OCI-Ly1 cells in a subcutaneous mouse xenograft model**. (a) Comparison of FBXL10 mRNA levels in 41 GCB DLBCL and 29 ABC DLBCL primary biopsies. FBXL10 mRNA levels were from Affymetrix Human Genome U133 plus 2.0 microarray data (GSE12195).^1^ (b) Tumor volumes were monitored and plotted for 7 weeks. The error bars denote S.E.M., n=6. (c) Numbers of tumor-bearing mice at the 7th week are shown.

**Figure S2 FBXL10 knockdown does not have any significant effect on cell cycles of** **OCI-Ly1 and** **SU-DHL-16 cells**. (a) Knockdown efficacy of FBXL10 by two independent shRNAs (shFBXL10#1 and shFBXL10#2) was analyzed by quantitative RT-PCR. The error bars denote S.E.M, n=3. (B-C) FBXL10 knockdown had no significant effect on the cell cycle of OCI-Ly1 (b) and SU-DHL-16 (c) cells. Cell phases were measured by FACS using propidium iodide staining.

**Figure S3** **The functional interaction between FBXL10 and BCL6.** (a) The mRNA levels of FBXL10 were positively correlated with BCL6 in 104 samples from ICGC malignant B cell lymphoma data set, values shown in the figure. (b) qRT-PCR analysis of BCL6 mRNA levels in eight different DLBCL cells and one control cells. (c) Western blots detection of BCL6 protein in different DLBCL cells and control cells. (d) FBXL10 interacted with BCL6 *in vivo*. HEK293T cells were cotransfected with pLenti-EF1-HA-FBXL10FL and pCMV3-Flag-BCL6 or pLenti-EF1-HA-

FBXL10ΔJmjC and pCMV3-Flag-BCL6. Western blotting assays with indicated antibodies were performed following IP with anti-FBXL10 antibody.

**Figure S4 The effect of FBXL10 knockdown on target genes in ABC DLBCL cells.** Expression levels of FBXL10 target genes (*DUSP6, HSPA8, PRDM10 and PRDX1*) in TMD8 (a) and U2932 (b) cells after FBXL10 knockdown. The mRNA levels were analyzed by quantitative RT-PCR. The error bars denote S.E.M., n=3.

**Supplementary Tables**

**Table S1 Primers used for RT-PCR and ChIP qPCR detection**

| **Primers** |  | **Sequence** |
| --- | --- | --- |
| *FBXL10* | forward | 5’- ATGTGATCCGGCCACCCCCCATC -3’ |
|  | reverse | 5’- TTGGAGATATTGGTCCAGCTGAG -3’ |
| *BCL6* | forward | 5’- GTTGTGGACACTTGCCGGAA -3’ |
|  | reverse | 5’- CTCTTCACGAGGAGGCTTGAT -3’ |
| *β-actin* | forward | 5’- CATGTACGTTGCTATCCAGGC -3’ |
|  | reverse | 5’- CTCCTTAATGTCACGCACGAT -3’ |
| *DUSP6* | forward | 5’- AGCTCAATCTGTCGATGAACG-3’ |
|  | reverse | 5’- GCGTCCTCTCGAAGTCCAG -3’ |
| *PRDM10* | forward | 5’- GGCTCGGAGCTGAAAGACTG -3’ |
|  | reverse | 5’- CGTCTCATCAGACAACTCAAACC -3’ |
| *HSPA8* | forward | 5’- ATGCCAAACGTCTGATTGGAC -3’ |
|  | reverse | 5’- AGCATCATTCACCACCATAAAGG -3’ |
| *PRDX1* | forward | 5’- CCACGGAGATCATTGCTTTCA -3’ |
|  | reverse | 5’- AGGTGTATTGACCCATGCTAGAT -3’ |
| *DUSP6* promoter | forward | 5’- TTCTAATCCCTCCCTCCA -3’ |
|  | reverse | 5’- GACGCTCGCTGTTTGTAT -3’ |
| *GAPDH* promoter | forward | 5’- GCCACATCGCTCAGACAC -3’ |
|  | reverse | 5’- CATACGACTGCAAAGACCC -3’ |

**Table S2** **List of antibodies, related to the materials and methods**

| **Antibody** | **Application** | **Dilution** | **Supplier** | **Cat. No.** |
| --- | --- | --- | --- | --- |
| **H3K36me2** | **IB**  **ChIP** | IB, 1:3000  ChIP, 5μl | Active motif [Carlsbad, CA] | #39255 |
| **H3K27me3** | **IB**  **ChIP** | IB, 1:1000  ChIP, 1:50 | Cell Signaling Technology [Danvers, MA] | #9733 |
| **H2AK119ub1** | **IB**  **ChIP** | IB, 1:1000  ChIP, 1:50 | Cell Signaling Technology [Danvers, MA] | #8240 |
| **Erk1/2** | **IB** | IB, 1:1000 | Cell Signaling Technology [Danvers, MA] | #9102 |
| **Phospho-Erk1/2** | **IB** | IB, 1:1000 | Cell Signaling Technology [Danvers, MA] | #4376 |
| **HA** | **IB**  **IP** | IB, 1:1000  IP, 1:50 | Cell Signaling Technology [Danvers, MA] | #3274 |
| **Anti-FLAG® M2 Magnetic Beads** | **IP** | IP, 40μl/ml | Sigma Aldrich [St. Louis, MO] | M8823 |
| **β-actin** | **IB** | IB, 1:20000 | Abcam [Cambridge, UK] | ab8826 |
| **BCL-6** | **IB** | IB, 1:200 | Santa Cruz [Santa Cruz, CA] | Sc-7388 |
| **DUSP6** | **IB** | IB, 1:500 | Abcam [Cambridge, UK] | Ab76310 |
| **Normal Rabbit IgG** | **ChIP** | ChIP, 5μl | Cell Signaling Technology [Danvers, MA] | #2729 |
| **Normal Moure IgG** | **ChIP** | ChIP, 5μl | Cell Signaling Technology [Danvers, MA] | #5415 |
| **Anti-Rabbit IgG HRP** | **IB** | IB, 1:5000 | Abcam [Cambridge, UK] | ab6721 |
| **Anti-Mouse IgG HRP** | **IB** | IB, 1:5000 | Abcam [Cambridge, UK] | ab6789 |

**Table S3** **List of genes that are up-regulated by FBXL10 knockdown in** **OCI-Ly1 cells**

The order of genes in the list is based simply on the mean of fold changes in expression that were induced by two shRNAs (shFBXL10#1 and shFBXL10#2) in OCI-Ly1 cells.

| **Gene Symbol** | **UniGene.ID** | **shControl FPKM** | **shFBXL10#1**  **FPKM** | **shFBXL10#2**  **FPKM** | |
| --- | --- | --- | --- | --- | --- |
| Upregulated genes | | | | |  |

| *COX7B* | 1349 | 269.07 | 404.03 | 402.96 |
| --- | --- | --- | --- | --- |
| *MRPL15* | 29088 | 55.42 | 83.19 | 84.77 |
| *RPUSD4* | 84881 | 7.75 | 11.64 | 11.94 |
| *CDC123* | 8872 | 75.82 | 113.98 | 117.09 |
| *RHOF* | 54509 | 9.87 | 15.33 | 14.85 |
| *MED26* | 9441 | 4.48 | 6.95 | 6.81 |
| *XPO1* | 7514 | 93.51 | 142.54 | 145.09 |
| *COPS4* | 51138 | 18.25 | 28.66 | 27.52 |
| *GINS1* | 9837 | 14.37 | 22.27 | 21.97 |
| *SRPRB* | 58477 | 13.81 | 21.2 | 21.35 |
| *SAR1A* | 56681 | 23.07 | 34.9 | 36.25 |
| *NOM1* | 64434 | 16.32 | 25.67 | 24.7 |
| *POLR2F* | 5435 | 37.31 | 56.63 | 58.65 |
| *ATR* | 545 | 14.48 | 21.78 | 22.97 |
| *DKC1* | 1736 | 57.39 | 89.07 | 88.37 |
| *NDUFS5* | 4725 | 227.09 | 341.91 | 362.09 |
| *EIF5A* | 1984 | 456.79 | 729.94 | 688.41 |
| *RAD54L* | 8438 | 7.39 | 11.91 | 11.06 |
| *INTS5* | 80789 | 5.67 | 9.03 | 8.62 |
| *SEC23B* | 10483 | 21.19 | 33.19 | 32.81 |
| *RARS2* | 57038 | 18.5 | 29.36 | 28.28 |
| *SACS* | 26278 | 1.78 | 2.85 | 2.71 |
| *POLE3* | 54107 | 45.47 | 72.31 | 69.81 |
| *GDI2* | 2665 | 204.61 | 307.55 | 333.24 |
| *FASTKD5* | 60493 | 8.07 | 12.96 | 12.32 |
| *PAICS* | 10606 | 140.74 | 223.18 | 218.01 |
| *CENPI* | 2491 | 5.23 | 8.18 | 8.23 |
| *EXOSC2* | 23404 | 29.33 | 47 | 45.06 |
| *GSG2* | 83903 | 7.71 | 12.37 | 11.85 |
| *DDX28* | 55794 | 4.72 | 7.61 | 7.22 |
| *C1QBP* | 708 | 107.73 | 176.91 | 162.28 |
| *MRPS7* | 51081 | 29.83 | 47.88 | 46.1 |
| *LARP4* | 113251 | 35.32 | 54.6 | 56.81 |
| *CENPJ* | 55835 | 10.28 | 16.03 | 16.5 |
| *RCOR1* | 23186 | 7.58 | 11.88 | 12.13 |
| *ACTR5* | 79913 | 5.81 | 9.16 | 9.25 |
| *NUP153* | 9972 | 23.84 | 39.39 | 36.18 |
| *CA8* | 767 | 12.1 | 18.12 | 20.24 |
| *DDX31* | 64794 | 6.45 | 10.52 | 9.95 |
| *NUP160* | 23279 | 35.33 | 55.39 | 56.81 |
| *SNHG12* | 85028 | 18.73 | 29 | 30.52 |
| *IKBKG* | 8517 | 11.55 | 18.64 | 18.11 |
| *FAM210A* | 125228 | 8.88 | 13.71 | 14.58 |
| *EIF2S1* | 1965 | 29.33 | 44.46 | 48.99 |
| *NSDHL* | 50814 | 12.82 | 21.02 | 19.83 |
| *EIF4A1* | 1973 | 312.3 | 488.52 | 506.85 |
| *SLC20A1* | 6574 | 13.06 | 22.07 | 19.61 |
| *NDUFS3* | 4722 | 35.95 | 58.55 | 56.2 |
| *SLC25A22* | 79751 | 6.71 | 11.17 | 10.25 |
| *DDI2* | 84301 | 7.01 | 11.61 | 10.78 |
| *PPA1* | 5464 | 134.96 | 210.82 | 220.57 |
| *NAA16* | 79612 | 12.38 | 18.56 | 21.03 |
| *LINC00987* | 1E+08 | 122.94 | 188.16 | 205.17 |
| *TRIM65* | 201292 | 8.01 | 13.32 | 12.31 |
| *MYH9* | 4627 | 112.5 | 172.46 | 187.63 |
| *FAM98B* | 283742 | 15.38 | 24.13 | 25.1 |
| *ACTN4* | 81 | 47.01 | 77.53 | 72.95 |
| *CYCS* | 54205 | 76.98 | 120.29 | 126.25 |
| *TTC27* | 55622 | 11.63 | 17.87 | 19.42 |
| *TCOF1* | 6949 | 35.9 | 55.99 | 59.37 |
| *TULP3* | 7289 | 4.66 | 7.23 | 7.76 |
| *LRMP* | 4033 | 235.41 | 403.69 | 356.01 |
| *MRPL20* | 55052 | 65.92 | 108.91 | 103.83 |
| *LLPH* | 84298 | 49.35 | 75.49 | 83.78 |
| *TM2D3* | 80213 | 5.53 | 9.34 | 8.51 |
| *DHX9* | 1660 | 93.96 | 147.94 | 156 |
| *FDX1* | 2230 | 16.06 | 25.68 | 26.33 |
| *C8orf33* | 65265 | 26.75 | 43.65 | 43 |
| *MRPL36* | 64979 | 24.59 | 40.38 | 39.29 |
| *SFXN1* | 94081 | 26.11 | 39.74 | 44.86 |
| *DYNLL1* | 8655 | 164.75 | 275 | 259.11 |
| *UBE2T* | 29089 | 47.75 | 82.16 | 72.88 |
| *TUBA1B* | 10376 | 744.31 | 1299.65 | 1119.18 |
| *NUP107* | 57122 | 30.94 | 47.39 | 53.34 |
| *PTMA* | 5757 | 1471.11 | 2213.81 | 2576.65 |
| *TOE1* | 114034 | 6.65 | 11.62 | 10.05 |
| *HNRNPH2* | 3188 | 5.81 | 9.48 | 9.47 |
| *NUDCD1* | 84955 | 7.48 | 11.46 | 12.96 |
| *TTC39C* | 125488 | 2.18 | 3.27 | 3.85 |
| *ANKRD13B* | 124930 | 2.85 | 4.76 | 4.55 |
| *UBQLN1* | 29979 | 40.29 | 66.25 | 65.44 |
| *PNPT1* | 87178 | 24.22 | 38.74 | 40.45 |
| *PLAA* | 9373 | 16.45 | 28.07 | 25.78 |
| *SNRNP25* | 79622 | 39.68 | 68.95 | 60.95 |
| *GOT2* | 2806 | 53.74 | 94.41 | 81.57 |
| *ARHGDIA* | 396 | 137.33 | 244.19 | 205.63 |
| *PRPF4* | 9128 | 18.43 | 30.68 | 29.71 |
| *DUS3L* | 56931 | 9.51 | 16.12 | 15.05 |
| *FASTKD1* | 79675 | 8.02 | 12.71 | 13.59 |
| *PGAM5* | 192111 | 26.69 | 46.15 | 41.52 |
| *DHDDS* | 79947 | 7.13 | 12.38 | 11.06 |
| *DDB1* | 1642 | 59.58 | 99.3 | 96.71 |
| *PPAT* | 5471 | 19.66 | 31.62 | 33.08 |
| *SKIV2L2* | 23517 | 33.44 | 52.22 | 57.89 |
| *TRAP1* | 10131 | 59.07 | 103.87 | 90.68 |
| *MRPL47* | 57129 | 41.13 | 68.93 | 66.73 |
| *IKZF1* | 10320 | 65.78 | 117.69 | 99.28 |
| *TXNDC17* | 84817 | 10.09 | 17.47 | 15.85 |
| *USP5* | 8078 | 22.42 | 39.95 | 34.09 |
| *ZC3HAV1* | 56829 | 12.41 | 18.97 | 22.02 |
| *MEF2D* | 4209 | 8.5 | 15.09 | 13.02 |
| *PSME2* | 5721 | 135.21 | 239.47 | 208.31 |
| *HNRNPAB* | 3182 | 172.74 | 312.66 | 259.54 |
| *MLEC* | 9761 | 19.14 | 31.73 | 31.68 |
| *TMEM141* | 85014 | 23.13 | 36.98 | 39.67 |
| *SSBP1* | 6742 | 194.42 | 293.17 | 351.18 |
| *TUBB6* | 84617 | 16.7 | 28.66 | 26.74 |
| *DYNC1H1* | 1778 | 23.43 | 39.23 | 38.5 |
| *BLOC1S3* | 388552 | 2.23 | 3.6 | 3.81 |
| *ZW10* | 9183 | 7.19 | 11.94 | 12.01 |
| *RELL2* | 285613 | 8.54 | 15.43 | 13.02 |
| *TBC1D14* | 57533 | 10.32 | 18.31 | 16.09 |
| *MSMO1* | 6307 | 17.42 | 28.67 | 29.4 |
| *CAND1* | 55832 | 26.1 | 43.89 | 43.13 |
| *RCC1* | 1104 | 46.56 | 71.78 | 83.47 |
| *BYSL* | 705 | 10.65 | 19.16 | 16.37 |
| *CEBPZ* | 10153 | 39.84 | 62.92 | 70.03 |
| *FAM216A* | 29902 | 8.2 | 14.21 | 13.16 |
| *CLTC* | 1213 | 53.38 | 89.59 | 88.72 |
| *PHRF1* | 57661 | 11.35 | 20.45 | 17.47 |
| *IPO7* | 10527 | 42.4 | 67.12 | 74.54 |
| *DHX15* | 1665 | 44.34 | 74.57 | 73.66 |
| *NOP9* | 161424 | 13.01 | 23.95 | 19.55 |
| *CENPV* | 201161 | 17.37 | 30.36 | 27.73 |
| *YWHAG* | 7532 | 89.88 | 148.26 | 152.53 |
| *TEC* | 7006 | 2.68 | 4.58 | 4.39 |
| *EXOSC3* | 51010 | 31.39 | 49.34 | 55.76 |
| *ADAM19* | 8728 | 6.76 | 11.71 | 10.95 |
| *RRP1* | 8568 | 33.61 | 62.33 | 50.45 |
| *TUBA1A* | 7846 | 40.36 | 69.22 | 66.27 |
| *SHQ1* | 55164 | 9.88 | 15.46 | 17.72 |
| *PSMB8* | 5696 | 43.79 | 78.39 | 68.75 |
| *UBE2N* | 7334 | 19.73 | 34.01 | 32.3 |
| *CCT7* | 10574 | 158.65 | 275.49 | 257.82 |
| *MSTO1* | 55154 | 7.53 | 13.33 | 12.01 |
| *CORO1C* | 23603 | 7.77 | 13.49 | 12.67 |
| *CAMKK2* | 10645 | 7.77 | 14.24 | 11.95 |
| *WDFY4* | 57705 | 12.32 | 22.58 | 18.96 |
| *FASTKD2* | 22868 | 7.88 | 13.26 | 13.31 |
| *CCT4* | 10575 | 85.79 | 143.9 | 145.77 |
| *PSMD8* | 5714 | 55.57 | 102.03 | 85.75 |
| *EXOSC7* | 23016 | 24.97 | 40.86 | 43.53 |
| *SLC6A6* | 6533 | 13.45 | 24.55 | 20.93 |
| *ATIC* | 471 | 68.03 | 115.85 | 114.24 |
| *DARS2* | 55157 | 10.18 | 16.58 | 17.87 |
| *NTMT1* | 28989 | 14.3 | 26.74 | 21.66 |
| *OAT* | 4942 | 10.81 | 17.42 | 19.2 |
| *CD63* | 967 | 29.38 | 53.65 | 46.08 |
| *HK1* | 3098 | 25.83 | 44.05 | 43.63 |
| *UTP3* | 57050 | 34.66 | 55.31 | 62.4 |
| *KARS* | 3735 | 90.68 | 165.93 | 142.11 |
| *ARL5A* | 26225 | 15.88 | 23.98 | 30.17 |
| *PKM* | 5315 | 331.26 | 561.27 | 568.88 |
| *DNAJC25* | 548645 | 2.21 | 3.63 | 3.91 |
| *TTLL12* | 23170 | 18.44 | 34.8 | 28.13 |
| *BZW2* | 28969 | 55.69 | 96.27 | 93.81 |
| *MAPKAPK3* | 7867 | 8.1 | 15.24 | 12.45 |
| *MRPL4* | 51073 | 45.9 | 88.29 | 68.62 |
| *XPO4* | 64328 | 6.82 | 12.72 | 10.6 |
| *LYPLA1* | 10434 | 58.4 | 101.86 | 97.86 |
| *STAG3L2* | 442582 | 12.49 | 21.78 | 20.94 |
| *DDX49* | 54555 | 16.94 | 32.21 | 25.81 |
| *CCDC73* | 493860 | 4.56 | 7.45 | 8.18 |
| *GCNT1* | 2650 | 12.6 | 20.92 | 22.27 |
| *ADSL* | 158 | 39.94 | 64.65 | 72.49 |
| *CORO7* | 79585 | 2.19 | 3.76 | 3.76 |
| *PMM2* | 5373 | 11.1 | 19.79 | 18.33 |
| *NOC2L* | 26155 | 34.17 | 62.42 | 55.02 |
| *TOMM22* | 56993 | 36.6 | 60.44 | 65.41 |
| *TCERG1* | 10915 | 37 | 55.84 | 71.47 |
| *UBE3C* | 9690 | 15.42 | 28.17 | 24.91 |
| *ISG20L2* | 81875 | 34.72 | 55.94 | 63.8 |
| *KPNA2* | 3838 | 127.14 | 197.54 | 241.06 |
| *PNO1* | 56902 | 17.38 | 29.65 | 30.33 |
| *NUDC* | 10726 | 95.87 | 167.9 | 162.97 |
| *RGS2* | 5997 | 13.61 | 22.38 | 24.64 |
| *NUP155* | 9631 | 15.69 | 26.74 | 27.51 |
| *C14orf169* | 79697 | 5.36 | 9.38 | 9.16 |
| *UAP1* | 6675 | 12.06 | 19.59 | 22.14 |
| *RRP1B* | 23076 | 22.38 | 38.53 | 39.01 |
| *EIF4G1* | 1981 | 134.7 | 233.23 | 233.52 |
| *NPM3* | 10360 | 18.4 | 35.3 | 28.46 |
| *GLRX* | 2745 | 15.03 | 25.64 | 26.47 |
| *NUP35* | 129401 | 11.89 | 20.4 | 20.85 |
| *RABL6* | 55684 | 34.81 | 66.05 | 54.72 |
| *RBM28* | 55131 | 35.33 | 53.29 | 69.3 |
| *RUVBL2* | 10856 | 43.66 | 77.25 | 74.27 |
| *NOLC1* | 9221 | 94.72 | 170.98 | 158.09 |
| *GINS4* | 84296 | 9.21 | 16.65 | 15.36 |
| *WDR3* | 10885 | 23.62 | 40.98 | 41.18 |
| *RBX1* | 9978 | 21.29 | 40.74 | 33.43 |
| *ELMO1* | 9844 | 24.57 | 45.89 | 39.75 |
| *SDCCAG3* | 10807 | 20.8 | 38.56 | 33.95 |
| *NOSIP* | 51070 | 13.47 | 26.81 | 20.15 |
| *PSME4* | 23198 | 34.68 | 63.17 | 57.84 |
| *FAM207A* | 85395 | 19.73 | 38.15 | 30.7 |
| *IDH3A* | 3419 | 26.91 | 46.73 | 47.18 |
| *C17orf97* | 400566 | 3.64 | 6.07 | 6.66 |
| *NUP62* | 23636 | 32.04 | 62.87 | 49.27 |
| *CTSC* | 1075 | 27.26 | 44.68 | 50.78 |
| *RRP15* | 51018 | 5.92 | 10.85 | 9.9 |
| *MTMR4* | 9110 | 7.86 | 15.3 | 12.25 |
| *SNX29P1* | 1.01E+08 | 119.22 | 227.58 | 190.33 |
| *SEC61B* | 10952 | 35.31 | 69.03 | 54.76 |
| *SH3D21* | 79729 | 3.28 | 5.64 | 5.86 |
| *SNHG15* | 285958 | 15.17 | 29.98 | 23.22 |
| *OTUD4* | 54726 | 15.28 | 25.22 | 28.37 |
| *LCP1* | 3936 | 319.31 | 517.4 | 602.59 |
| *MORF4L2* | 9643 | 72.28 | 127.76 | 125.81 |
| *NIP7* | 51388 | 20.24 | 33.63 | 37.42 |
| *BCCIP* | 56647 | 33.45 | 51.89 | 65.55 |
| *ILF2* | 3608 | 99.68 | 183.11 | 166.87 |
| *RELT* | 84957 | 5.63 | 11.31 | 8.49 |
| *UTP15* | 84135 | 7.32 | 12.87 | 12.88 |
| *RRP12* | 23223 | 10.05 | 17.79 | 17.59 |
| *PSMD3* | 5709 | 47.23 | 91.46 | 75.17 |
| *CAD* | 790 | 17.19 | 34.3 | 26.43 |
| *SLC43A3* | 29015 | 41.28 | 78.42 | 67.45 |
| *XRCC5* | 7520 | 132.71 | 223.48 | 245.53 |
| *SIT1* | 27240 | 29.43 | 59.6 | 44.42 |
| *NAT10* | 55226 | 26.69 | 45.13 | 49.39 |
| *MARS2* | 92935 | 2.43 | 4.16 | 4.45 |
| *EEA1* | 8411 | 7.57 | 11.53 | 15.3 |
| *GLA* | 2717 | 6.31 | 11.26 | 11.11 |
| *SELK* | 58515 | 31.03 | 60.34 | 49.72 |
| *EDC4* | 23644 | 9.93 | 20.15 | 15.09 |
| *G6PD* | 2539 | 20.46 | 40.05 | 32.68 |
| *PLEKHF2* | 79666 | 39.77 | 71.75 | 69.63 |
| *NOL8* | 55035 | 22.8 | 41.18 | 39.88 |
| *PGAM1* | 5223 | 108.78 | 207.56 | 179.49 |
| *PWP2* | 5822 | 11.46 | 22.13 | 18.65 |
| *PCYT2* | 5833 | 8.81 | 18.07 | 13.29 |
| *ATP5A1* | 498 | 128.7 | 214.22 | 244.1 |
| *TPRKB* | 51002 | 30.53 | 48.84 | 60.08 |
| *MTF2* | 22823 | 24.4 | 40.29 | 46.8 |
| *PES1* | 23481 | 33.44 | 60.49 | 58.94 |
| *FASTKD3* | 79072 | 5.57 | 10.43 | 9.47 |
| *PSMD12* | 5718 | 19.03 | 29.65 | 38.37 |
| *QTRT2* | 79691 | 11.18 | 19.39 | 20.62 |
| *DDX24* | 57062 | 72.66 | 125.33 | 134.88 |
| *PDE12* | 201626 | 8 | 15.1 | 13.61 |
| *APEH* | 327 | 13.73 | 27.97 | 21.34 |
| *FBLN1* | 2192 | 2.48 | 4.68 | 4.24 |
| *UBAP2L* | 9898 | 56.5 | 101.07 | 102.15 |
| *TFR2* | 7036 | 4.39 | 9.09 | 6.71 |
| *TTF2* | 8458 | 21.43 | 36.52 | 40.61 |
| *POLR1B* | 84172 | 9.02 | 16.16 | 16.33 |
| *PDCD2L* | 84306 | 11.11 | 19.92 | 20.11 |
| *UTP14A* | 10813 | 27.01 | 47.08 | 50.28 |
| *AIM2* | 9447 | 7.1 | 13.06 | 12.57 |
| *HLA-A* | 3105 | 22.04 | 46.3 | 33.36 |
| *URB1* | 9875 | 4.84 | 9.14 | 8.36 |
| *PIEZO1* | 9780 | 2.25 | 4.09 | 4.05 |
| *YRDC* | 79693 | 5.07 | 8.17 | 10.18 |
| *ENO1* | 2023 | 277.54 | 495.61 | 509.16 |
| *TMSB10* | 9168 | 517.56 | 965.11 | 909.07 |
| *DHX33* | 56919 | 8.5 | 17.13 | 13.67 |
| *HSPB1* | 3315 | 11.61 | 20.89 | 21.18 |
| *STRIP2* | 57464 | 4.1 | 7.16 | 7.71 |
| *ZWILCH* | 55055 | 20.22 | 35.52 | 37.83 |
| *HDAC9* | 9734 | 14.11 | 27.97 | 23.22 |
| *WDR75* | 84128 | 22.52 | 37.65 | 44.07 |
| *CACYBP* | 27101 | 50.76 | 86.72 | 97.73 |
| *SRRT* | 51593 | 66.97 | 116.16 | 127.27 |
| *CFAP47* | 286464 | 2.18 | 3.92 | 4.01 |
| *THRIL* | 1.03E+08 | 23.32 | 47.14 | 37.7 |
| *SHCBP1* | 79801 | 26.42 | 51.87 | 44.27 |
| *SNAPC4* | 6621 | 5.99 | 10.79 | 11.01 |
| *BIK* | 638 | 12.43 | 23.34 | 21.94 |
| *RAP1B* | 5908 | 100.77 | 158.38 | 208.96 |
| *TEFM* | 79736 | 4.23 | 7.05 | 8.37 |
| *SPRYD4* | 283377 | 3.49 | 6.16 | 6.59 |
| *FANCB* | 2187 | 3.41 | 6.3 | 6.16 |
| *PDIA6* | 10130 | 71.92 | 139.65 | 123.53 |
| *TIMM8B* | 26521 | 78.66 | 144.7 | 143.19 |
| *NOP56* | 10528 | 89.55 | 163.71 | 164.71 |
| *HNRNPA2B1* | 3181 | 483.86 | 756.37 | 1019.04 |
| *FBXO41* | 150726 | 7.34 | 14.57 | 12.37 |
| *PMPCA* | 23203 | 17.21 | 33.06 | 30.16 |
| *SHOX2* | 6474 | 1.27 | 2.44 | 2.23 |
| *NUP205* | 23165 | 30.01 | 54.33 | 56.17 |
| *ORC1* | 4998 | 16 | 31.68 | 27.25 |
| *RFC3* | 5983 | 32.9 | 58.98 | 62.31 |
| *CAPRIN1* | 4076 | 70.32 | 127.71 | 131.58 |
| *TMEM177* | 80775 | 3.53 | 7.01 | 6.02 |
| *TARDBP* | 23435 | 47.95 | 84.71 | 92.31 |
| *LOC102724428* | 1.03E+08 | 1.04 | 1.75 | 2.09 |
| *UTP4* | 84916 | 29.55 | 58.32 | 50.79 |
| *HEATR1* | 55127 | 15.81 | 28.37 | 30.08 |
| *PRMT5-AS1* | 1.01E+08 | 6.77 | 12.63 | 12.43 |
| *NOCT* | 25819 | 3.37 | 5.68 | 6.8 |
| *PA2G4* | 5036 | 200.06 | 358.31 | 382.69 |
| *SCO1* | 6341 | 13.63 | 22.18 | 28.35 |
| *EEF1E1* | 9521 | 26.81 | 49.09 | 50.31 |
| *WDR36* | 134430 | 13.44 | 24.28 | 25.59 |
| *DNAAF5* | 54919 | 6.33 | 13.28 | 10.22 |
| *PUM3* | 9933 | 53.72 | 95.58 | 104.54 |
| *NLE1* | 54475 | 3.4 | 7.08 | 5.59 |
| *COX7C* | 1350 | 89.02 | 159.33 | 172.79 |
| *LTV1* | 84946 | 28.05 | 46.76 | 57.98 |
| *BRIX1* | 55299 | 49.81 | 80.42 | 105.66 |
| *HPRT1* | 3251 | 41.74 | 73.79 | 82.33 |
| *SPTBN2* | 6712 | 8.71 | 18.52 | 14.06 |
| *CCDC78* | 124093 | 5.79 | 11.83 | 9.87 |
| *DNTTIP2* | 30836 | 38.15 | 66.59 | 76.44 |
| *UGDH* | 7358 | 11.7 | 23.11 | 20.8 |
| *WDR46* | 9277 | 26.1 | 52.07 | 45.93 |
| *ALYREF* | 10189 | 95.27 | 197.43 | 160.44 |
| *PPP2R1B* | 5519 | 32.31 | 57.54 | 64.03 |
| *BOLA2* | 552900 | 37.47 | 81.64 | 59.58 |
| *MCM10* | 55388 | 15.29 | 30.9 | 26.78 |
| *GEMIN6* | 79833 | 17.14 | 32.02 | 32.65 |
| *WDR43* | 23160 | 29.51 | 49.26 | 62.13 |
| *MRPS12* | 6183 | 11 | 24.58 | 17.01 |
| *EHD1* | 10938 | 41.87 | 92.06 | 66.38 |
| *UCK2* | 7371 | 6.91 | 14.31 | 11.85 |
| *SUV39H2* | 79723 | 10.19 | 18.48 | 20.12 |
| *DBNL* | 28988 | 51.99 | 112.09 | 84.85 |
| *MS4A1* | 931 | 185.07 | 370.47 | 330.81 |
| *SLC4A7* | 9497 | 2.36 | 4.08 | 4.87 |
| *KRTCAP3* | 200634 | 4.39 | 8.05 | 8.6 |
| *DFFB* | 1677 | 5.62 | 10.9 | 10.42 |
| *DDX18* | 8886 | 29.98 | 54.37 | 59.4 |
| *ERCC6L* | 54821 | 5.1 | 7.82 | 11.54 |
| *EMG1* | 10436 | 28.48 | 55.39 | 52.79 |
| *PPIH* | 10465 | 36.46 | 59.71 | 78.8 |
| *DCAF13* | 25879 | 26.05 | 46.75 | 52.23 |
| *RPP40* | 10799 | 4.6 | 9.62 | 7.86 |
| *MRPL12* | 6182 | 34.44 | 70.59 | 60.51 |
| *GPI* | 2821 | 45.59 | 93.35 | 80.45 |
| *MSN* | 4478 | 52.42 | 92.92 | 106.95 |
| *MKNK2* | 2872 | 8.66 | 18.91 | 14.18 |
| *PFKP* | 5214 | 45.97 | 95.91 | 79.81 |
| *TPP2* | 7174 | 37.07 | 73.03 | 68.7 |
| *PPARGC1B* | 133522 | 2.91 | 5.82 | 5.31 |
| *AAMP* | 14 | 19.85 | 41.73 | 34.2 |
| *PPHLN1* | 51535 | 39.19 | 79.78 | 70.15 |
| *PSMD13* | 5719 | 47.42 | 90.82 | 90.68 |
| *IFRD2* | 7866 | 9.64 | 20.37 | 16.53 |
| *NCL* | 4691 | 494.05 | 856.7 | 1035.99 |
| *B4GALNT3* | 283358 | 1.15 | 2.34 | 2.07 |
| *LSMEM2* | 132228 | 6.27 | 11.11 | 12.94 |
| *TMC8* | 147138 | 5.99 | 12.95 | 10.03 |
| *HNRNPR* | 10236 | 39.44 | 68.58 | 82.75 |
| *DOK3* | 79930 | 27.66 | 60.46 | 45.69 |
| *BOLA2-SMG1P6* | 1.07E+08 | 8.38 | 16.91 | 15.26 |
| *SFXN2* | 118980 | 6.23 | 14.32 | 9.65 |
| *ZNF576* | 79177 | 4.87 | 10.14 | 8.6 |
| *CLIC1* | 1192 | 91.75 | 203.72 | 149.38 |
| *ZNF668* | 79759 | 2.12 | 4.6 | 3.56 |
| *MZB1* | 51237 | 75.72 | 176.08 | 115.41 |
| *YIF1A* | 10897 | 10.67 | 23.97 | 17.16 |
| *ATP5G1* | 516 | 42.79 | 90.28 | 74.69 |
| *MAGED1* | 9500 | 17.78 | 40.74 | 27.85 |
| *PRR13* | 54458 | 65.04 | 125.49 | 125.52 |
| *FAAP100* | 80233 | 2.42 | 5.5 | 3.84 |
| *UBA1* | 7317 | 40.27 | 90.13 | 65.33 |
| *HSPA4L* | 22824 | 7.37 | 12.44 | 16.04 |
| *MRPL52* | 122704 | 14.48 | 30.79 | 25.17 |
| *EBPL* | 84650 | 5.29 | 10.18 | 10.28 |
| *GCDH* | 2639 | 9.45 | 20.18 | 16.37 |
| *SASH3* | 54440 | 19.18 | 36.88 | 37.37 |
| *COCH* | 1690 | 14.18 | 29.86 | 25.14 |
| *HPS6* | 79803 | 2.25 | 4.38 | 4.35 |
| *RIPK3* | 11035 | 3.29 | 7.36 | 5.41 |
| *DNAJB11* | 51726 | 57.71 | 130.46 | 94.42 |
| *TMED9* | 54732 | 30.41 | 68.33 | 50.26 |
| *ATP1B3* | 483 | 30.47 | 58.87 | 59.97 |
| *NME1* | 4830 | 129.66 | 288.44 | 217.59 |
| *HNRNPA3* | 220988 | 112.85 | 202.92 | 238.21 |
| *AKAP1* | 8165 | 13.68 | 28.65 | 24.83 |
| *PDCD11* | 22984 | 15 | 27.84 | 30.81 |
| *COPRS* | 55352 | 9.36 | 22.53 | 14.08 |
| *CCNC* | 892 | 32.82 | 55.96 | 72.55 |
| *TRIM21* | 6737 | 8.72 | 15.84 | 18.32 |
| *PRMT6* | 55170 | 6 | 12.27 | 11.24 |
| *LOC100505716* | 1.01E+08 | 2.23 | 4.78 | 3.96 |
| *PPAN* | 56342 | 26.65 | 57.55 | 46.9 |
| *MRPS30* | 10884 | 14.07 | 24.91 | 30.28 |
| *ITGA4* | 3676 | 15.28 | 30.15 | 29.87 |
| *TMEM102* | 284114 | 2.25 | 4.91 | 3.94 |
| *FPGS* | 2356 | 11.56 | 25.53 | 19.94 |
| *KPNA3* | 3839 | 17.97 | 32.51 | 38.2 |
| *FTSJ3* | 117246 | 24.84 | 44.43 | 53.36 |
| *CSK* | 1445 | 57.49 | 120.11 | 106.47 |
| *LPAR5* | 57121 | 2.58 | 5.72 | 4.45 |
| *ITPRIPL1* | 150771 | 4.53 | 9.07 | 8.79 |
| *ABRACL* | 58527 | 21.12 | 37.36 | 45.94 |
| *ELAC2* | 60528 | 15.38 | 33.77 | 26.95 |
| *CPNE3* | 8895 | 17.26 | 30.71 | 37.44 |
| *PPIF* | 10105 | 20.7 | 39.78 | 42 |
| *SHB* | 6461 | 0.46 | 0.9 | 0.92 |
| *ANXA2* | 302 | 25.59 | 45.28 | 56.02 |
| *ATP6V1C2* | 245973 | 20.41 | 45.13 | 35.7 |
| *NOL11* | 25926 | 28.39 | 56.01 | 56.57 |
| *LRRK1* | 79705 | 12.06 | 28.62 | 19.21 |
| *MEST* | 4232 | 5.18 | 9.87 | 10.68 |
| *TAGLN2* | 8407 | 47.26 | 111.39 | 76.39 |
| *BAZ1A* | 11177 | 20.46 | 37.96 | 43.34 |
| *ZNF804A* | 91752 | 9.62 | 17.93 | 20.3 |
| *ZYX* | 7791 | 7.8 | 17.11 | 13.89 |
| *DDX20* | 11218 | 9.86 | 18.28 | 20.92 |
| *RPS26* | 6231 | 238.05 | 507.43 | 439.27 |
| *GM2A* | 2760 | 23.45 | 52.8 | 40.68 |
| *NAA15* | 80155 | 36.34 | 65.87 | 79.14 |
| *RHPN2* | 85415 | 1.88 | 3.7 | 3.82 |
| *SRXN1* | 140809 | 10.39 | 18.39 | 23.19 |
| *RASSF2* | 9770 | 32.15 | 78.84 | 49.88 |
| *SNX29P2* | 440352 | 26.71 | 57.12 | 50.21 |
| *SOX4* | 6659 | 1.08 | 2.05 | 2.29 |
| *LOC100506071* | 1.01E+08 | 6.72 | 14.83 | 12.24 |
| *MCM4* | 4173 | 69.28 | 165.85 | 113.28 |
| *IDE* | 3416 | 12.24 | 24.92 | 24.42 |
| *KMO* | 8564 | 2.1 | 5 | 3.47 |
| *KCTD12* | 115207 | 1.15 | 2.47 | 2.18 |
| *CDC25A* | 993 | 11.65 | 24.04 | 23.1 |
| *NLN* | 57486 | 5.65 | 13.28 | 9.59 |
| *DDIAS* | 220042 | 11.19 | 22.53 | 22.78 |
| *ODC1* | 4953 | 155.37 | 333.69 | 295.57 |
| *TEX10* | 54881 | 11.59 | 21.29 | 25.67 |
| *MCOLN2* | 255231 | 14.57 | 27.51 | 31.54 |
| *UTP11* | 51118 | 22 | 44.88 | 44.33 |
| *EIF3J* | 8669 | 29.94 | 50.46 | 71.05 |
| *EIF5B* | 9669 | 59.86 | 103.34 | 139.63 |
| *POLR3G* | 10622 | 9.62 | 16.37 | 22.68 |
| *ABCE1* | 6059 | 46.93 | 85.36 | 105.3 |
| *SPN* | 6693 | 11.92 | 24.43 | 24.13 |
| *TWF1* | 5756 | 17.29 | 38.87 | 31.57 |
| *PLOD1* | 5351 | 4.88 | 10.81 | 9.09 |
| *EIF5AL1* | 143244 | 1.05 | 2.12 | 2.17 |
| *EARS2* | 124454 | 5.49 | 11.75 | 10.72 |
| *SNORA40* | 677822 | 60.61 | 116.68 | 131.65 |
| *ATAD3A* | 55210 | 27.94 | 64.42 | 50.13 |
| *ASB2* | 51676 | 16.04 | 30.11 | 35.66 |
| *CCT6A* | 908 | 141.15 | 274.02 | 305.03 |
| *PHB* | 5245 | 41.02 | 90.36 | 78.06 |
| *SLC2A6* | 11182 | 2.41 | 5.02 | 4.88 |
| *AMD1* | 262 | 26.14 | 52.04 | 55.5 |
| *CD84* | 8832 | 10.22 | 26.74 | 15.37 |
| *ACAD9* | 28976 | 19.58 | 41.39 | 39.32 |
| *PSMA3* | 5684 | 63.63 | 127.27 | 135.15 |
| *C11orf98* | 1.02E+08 | 107.64 | 224.31 | 219.82 |
| *FEN1* | 2237 | 73 | 165.49 | 135.83 |
| *NDUFB2* | 4708 | 26.33 | 65.42 | 43.37 |
| *PYCRL* | 65263 | 3.94 | 10.21 | 6.08 |
| *DPP3* | 10072 | 13.65 | 34.38 | 22.06 |
| *SNHG4* | 724102 | 11.31 | 20.83 | 25.99 |
| *CCDC167* | 154467 | 15.01 | 35.49 | 26.67 |
| *RRP9* | 9136 | 12.04 | 25.15 | 24.73 |
| *GTPBP4* | 23560 | 42.19 | 80.27 | 94.53 |
| *AGRN* | 375790 | 1.14 | 2.97 | 1.76 |
| *SNRPA1* | 6627 | 65.24 | 131.13 | 139.71 |
| *POLR1A* | 25885 | 10.97 | 22.32 | 23.26 |
| *MRTO4* | 51154 | 32.13 | 65.03 | 68.51 |
| *PPP1R14B* | 26472 | 109.25 | 247.21 | 207.71 |
| *PIGW* | 284098 | 4.66 | 7.7 | 11.71 |
| *POLR2L* | 5441 | 41.83 | 110.16 | 64.11 |
| *RPP25* | 54913 | 4.78 | 11.58 | 8.34 |
| *HSPD1* | 3329 | 306.03 | 529.04 | 748.12 |
| *TIMM23* | 1E+08 | 38.38 | 77.55 | 82.7 |
| *VARS* | 7407 | 12.91 | 30.99 | 23.01 |
| *MYD88* | 4615 | 11.2 | 25.07 | 21.84 |
| *DHCR24* | 1718 | 18.07 | 41.84 | 33.89 |
| *TCL1A* | 8115 | 35.3 | 82.98 | 65 |
| *MACC1* | 346389 | 5.89 | 11.97 | 12.77 |
| *TCL1B* | 9623 | 112.14 | 271.03 | 200.03 |
| *ESRP2* | 80004 | 3.88 | 8.07 | 8.23 |
| *RPS6KA4* | 8986 | 9.25 | 23.96 | 14.94 |
| *FUBP1* | 8880 | 77.88 | 174.35 | 153.65 |
| *TSR1* | 55720 | 22.84 | 45.65 | 50.62 |
| *PLD6* | 201164 | 2.46 | 5.14 | 5.23 |
| *CD24* | 1E+08 | 110.81 | 245.56 | 221.66 |
| *NUMBL* | 9253 | 2.75 | 6.26 | 5.34 |
| *FHOD1* | 29109 | 4.84 | 12.99 | 7.49 |
| *BAG1* | 573 | 24.26 | 54.75 | 47.97 |
| *EIF3B* | 8662 | 63.25 | 135.26 | 132.8 |
| *TPI1* | 7167 | 162.7 | 374.99 | 314.63 |
| *PRMT5* | 10419 | 21.94 | 50.99 | 42.08 |
| *RBM12* | 10137 | 12.33 | 26.24 | 26.15 |
| *SURF6* | 6838 | 15.83 | 33.88 | 33.47 |
| *C5orf30* | 90355 | 2.56 | 6 | 4.9 |
| *HMGCR* | 3156 | 16.19 | 31.21 | 37.9 |
| *NDC1* | 55706 | 12.93 | 29.27 | 25.93 |
| *RARS* | 5917 | 27.25 | 54.45 | 61.94 |
| *GNL3* | 26354 | 79.29 | 154.08 | 184.94 |
| *NOTCH1* | 4851 | 1.41 | 3.62 | 2.42 |
| *ATRIP* | 84126 | 4.57 | 11.06 | 8.52 |
| *SCARB1* | 949 | 1.69 | 4.2 | 3.05 |
| *BRI3BP* | 140707 | 18 | 44.75 | 32.48 |
| *TELO2* | 9894 | 4.6 | 12.36 | 7.39 |
| *OAS3* | 4940 | 10.27 | 25.43 | 18.7 |
| *PTGER4* | 5734 | 1.62 | 3.61 | 3.36 |
| *TCP1* | 6950 | 88.68 | 172.43 | 209.6 |
| *PLCG1-AS1* | 1.02E+08 | 4.28 | 8.84 | 9.6 |
| *TUBB* | 203068 | 296.04 | 685.8 | 592.19 |
| *ANXA6* | 309 | 10.99 | 28.13 | 19.35 |
| *HMGCS1* | 3157 | 26.51 | 62.14 | 52.69 |
| *PTGDR2* | 11251 | 2.56 | 6.38 | 4.71 |
| *RAB3A* | 5864 | 2.5 | 5.86 | 4.98 |
| *FDFT1* | 2222 | 42.67 | 85 | 100.08 |
| *SLC38A5* | 92745 | 4.29 | 11.74 | 6.88 |
| *CORO1A* | 11151 | 85.58 | 203.14 | 168.47 |
| *TUBB4A* | 10382 | 2.1 | 4.62 | 4.56 |
| *CHORDC1* | 26973 | 23.84 | 37.79 | 66.46 |
| *DEF8* | 54849 | 28.98 | 77 | 49.79 |
| *G3BP1* | 10146 | 46.56 | 96.06 | 107.76 |
| *NAPSB* | 256236 | 26.6 | 71.36 | 45.15 |
| *SYNCRIP* | 10492 | 108.78 | 213.17 | 264.15 |
| *SRSF2* | 6427 | 79.55 | 175.73 | 173.41 |
| *GRPEL1* | 80273 | 7.31 | 12.85 | 19.26 |
| *NUP93* | 9688 | 17.86 | 40.82 | 37.82 |
| *SORD* | 6652 | 15.52 | 33.09 | 35.46 |
| *TOMM40* | 10452 | 42.85 | 106.28 | 83.79 |
| *CCT5* | 22948 | 67.22 | 137.96 | 160.3 |
| *ACAA1* | 30 | 7.94 | 21.42 | 13.83 |
| *LRP4* | 4038 | 2.06 | 3.91 | 5.24 |
| *ALDOA* | 226 | 223.05 | 533.69 | 458.11 |
| *TXN* | 7295 | 44.74 | 91.98 | 107.49 |
| *PEG10* | 23089 | 24.84 | 71.48 | 39.31 |
| *RRS1* | 23212 | 23.39 | 51.54 | 52.87 |
| *JMJD4* | 65094 | 2.17 | 5.29 | 4.4 |
| *NDUFAF3* | 25915 | 9.66 | 25.84 | 17.33 |
| *POMP* | 51371 | 29.65 | 71.04 | 61.54 |
| *UCHL5* | 51377 | 33.61 | 68.04 | 82.51 |
| *DDX10* | 1662 | 16.68 | 32.35 | 42.37 |
| *LRP8* | 7804 | 9.18 | 21.65 | 19.54 |
| *BTK* | 695 | 37.54 | 86.62 | 82.54 |
| *URB2* | 9816 | 3.22 | 7.28 | 7.3 |
| *CANX* | 821 | 83.4 | 181.26 | 196.42 |
| *FAM195A* | 84331 | 5.38 | 13.14 | 11.23 |
| *BOP1* | 23246 | 23.93 | 64.73 | 43.7 |
| *AMIGO2* | 347902 | 2.63 | 5.93 | 6.02 |
| *SNORD22* | 9304 | 25.17 | 58.99 | 55.38 |
| *HGS* | 9146 | 15.61 | 41.63 | 29.6 |
| *PDIA5* | 10954 | 3.2 | 7.72 | 6.9 |
| *GPATCH4* | 54865 | 30.78 | 65.94 | 74.81 |
| *TOP1* | 7150 | 80.52 | 184.23 | 184.18 |
| *PSMB6* | 5694 | 34.47 | 88.21 | 69.61 |
| *MTCH2* | 23788 | 31.75 | 70.91 | 75.07 |
| *DPH2* | 1802 | 7.05 | 17.44 | 15.02 |
| *SOD2* | 6648 | 16.44 | 43.44 | 32.45 |
| *FAM81A* | 145773 | 32.23 | 72.87 | 76.19 |
| *SPNS2* | 124976 | 2.84 | 7.39 | 5.77 |
| *WDR77* | 79084 | 14.47 | 36.78 | 30.28 |
| *EIF4E2* | 9470 | 15.01 | 33.24 | 36.4 |
| *ARPC3* | 10094 | 57.9 | 131.73 | 136.97 |
| *SLC25A17* | 10478 | 9.01 | 18.93 | 22.9 |
| *ICOSLG* | 23308 | 8.23 | 21.66 | 16.7 |
| *CERKL* | 375298 | 4.64 | 11.08 | 10.63 |
| *PFDN6* | 10471 | 87.51 | 212.44 | 199.08 |
| *SERHL2* | 253190 | 5.82 | 13.04 | 14.33 |
| *SLC29A2* | 3177 | 9.27 | 23.15 | 20.45 |
| *TIMM8A* | 1678 | 4.99 | 12.34 | 11.14 |
| *CCT3* | 7203 | 122.22 | 291.53 | 283.61 |
| *PPIB* | 5479 | 130.73 | 341.43 | 274.18 |
| *GNL2* | 29889 | 35.48 | 81.75 | 87.01 |
| *RRP7A* | 27341 | 13.75 | 33.82 | 31.74 |
| *GCSH* | 2653 | 20.65 | 46.04 | 52.55 |
| *NCF1* | 653361 | 65.24 | 203.46 | 108.02 |
| *BST2* | 684 | 33.12 | 76.4 | 81.98 |
| *KCNK6* | 9424 | 1.03 | 2.71 | 2.22 |
| *COA4* | 51287 | 35.17 | 98.95 | 69.53 |
| *C1orf122* | 127687 | 4.9 | 13.4 | 10.1 |
| *LRRC59* | 55379 | 37.06 | 87.76 | 90 |
| *FARSA* | 2193 | 28.47 | 78.11 | 58.61 |
| *TBRG4* | 9238 | 13.17 | 36.87 | 26.53 |
| *VWCE* | 220001 | 1.96 | 5.6 | 3.84 |
| *NEK5* | 341676 | 0.71 | 1.79 | 1.63 |
| *DLEU2L* | 79469 | 5.35 | 9.78 | 16 |
| *CIITA* | 4261 | 6.17 | 18.67 | 11.07 |
| *ATAD3B* | 83858 | 8.78 | 23.59 | 18.91 |
| *CCDC85B* | 11007 | 9.79 | 27.78 | 19.67 |
| *ACY1* | 95 | 1.75 | 4.82 | 3.67 |
| *IFI30* | 10437 | 31.91 | 83.24 | 71.66 |
| *FAM166A* | 401565 | 26.97 | 73.84 | 57.8 |
| *CCT2* | 10576 | 96.91 | 222.85 | 251.46 |
| *SPIRE1* | 56907 | 0.39 | 0.87 | 1.04 |
| *IMPDH1* | 3614 | 10.73 | 27.32 | 25.24 |
| *VCP* | 7415 | 66.98 | 170.17 | 158.51 |
| *HAUS7* | 55559 | 7.07 | 22.57 | 12.2 |
| *FAH* | 2184 | 3.81 | 11.91 | 6.83 |
| *UTP20* | 27340 | 8.26 | 20.86 | 19.82 |
| *NT5DC2* | 64943 | 10.66 | 31.78 | 20.9 |
| *TIMM17A* | 10440 | 18.47 | 48.16 | 43.98 |
| *SNX22* | 79856 | 15.48 | 45.59 | 31.74 |
| *GRWD1* | 83743 | 8.16 | 22.04 | 18.99 |
| *PSMD1* | 5707 | 38.55 | 92.72 | 101.22 |
| *GPR75* | 10936 | 0.97 | 2.59 | 2.29 |
| *SCFD2* | 152579 | 3.83 | 11.24 | 8.03 |
| *PPRC1* | 23082 | 8.02 | 19.64 | 20.72 |
| *SLIRP* | 81892 | 53.06 | 125.7 | 141.6 |
| *PDIA3* | 2923 | 126.45 | 334.21 | 305.69 |
| *C17orf96* | 1E+08 | 2.05 | 5.65 | 4.74 |
| *FKBP11* | 51303 | 13.36 | 38.34 | 29.91 |
| *PLCD4* | 84812 | 0.73 | 1.59 | 2.14 |
| *DNAJB1* | 3337 | 18.37 | 43.78 | 50.26 |
| *SAPCD2* | 89958 | 8.62 | 22.79 | 21.36 |
| *GSTO1* | 9446 | 2.2 | 5.87 | 5.44 |
| *BMS1P17* | 1.01E+08 | 7.48 | 18.43 | 20.04 |
| *MRM1* | 79922 | 1.73 | 5.4 | 3.57 |
| *GAMT* | 2593 | 4.47 | 14.91 | 8.27 |
| *BOLA3* | 388962 | 24.01 | 63.65 | 60.87 |
| *NOP2* | 4839 | 29.7 | 76.49 | 77.97 |
| *KBTBD8* | 84541 | 2.74 | 5.16 | 9.09 |
| *EAF2* | 55840 | 20.88 | 51.39 | 57.28 |
| *IPO4* | 79711 | 7.46 | 23.1 | 15.78 |
| *METTL1* | 4234 | 4.07 | 11.05 | 10.25 |
| *GEMIN4* | 50628 | 6.4 | 19.69 | 13.86 |
| *TSACC* | 128229 | 2.63 | 6.66 | 7.16 |
| *NOL6* | 65083 | 9.07 | 26.07 | 21.7 |
| *TRMT10C* | 54931 | 10.79 | 23.1 | 33.85 |
| *INF2* | 64423 | 2.34 | 7.14 | 5.25 |
| *VPS9D1-AS1* | 1E+08 | 5.99 | 18.57 | 13.22 |
| *TUBB3* | 10381 | 31 | 100.1 | 64.49 |
| *TLR9* | 54106 | 1.84 | 5.46 | 4.34 |
| *MPEG1* | 219972 | 1.31 | 4.79 | 2.25 |
| *CSE1L* | 1434 | 46.84 | 126.75 | 126.49 |
| *KIAA0754* | 643314 | 0.84 | 2.08 | 2.47 |
| *CD180* | 4064 | 11.08 | 34.99 | 25.31 |
| *ALDH1B1* | 219 | 1.44 | 3.05 | 4.81 |
| *CRELD2* | 79174 | 25.87 | 91.48 | 49.74 |
| *CD72* | 971 | 42.64 | 140.29 | 92.63 |
| *SEC61G* | 23480 | 27.39 | 72.08 | 77.98 |
| *TUBB4B* | 10383 | 80.91 | 242.85 | 200.48 |
| *FTL* | 2512 | 266.45 | 952.17 | 516.09 |
| *PDIA4* | 9601 | 33.29 | 106.22 | 77.39 |
| *HEG1* | 57493 | 1.2 | 3.11 | 3.52 |
| *DGKG* | 1608 | 0.62 | 1.53 | 1.9 |
| *MYBBP1A* | 10514 | 18.64 | 56.48 | 46.79 |
| *OPN3* | 23596 | 0.72 | 2.37 | 1.62 |
| *BAG2* | 9532 | 13.36 | 35.96 | 38.38 |
| *ISOC2* | 79763 | 13.6 | 48.26 | 27.48 |
| *CTTN* | 2017 | 2.65 | 9.86 | 4.93 |
| *SNORD3A* | 780851 | 23 | 41.18 | 87.33 |
| *MFNG* | 4242 | 13.72 | 47.16 | 29.87 |
| *FASN* | 2194 | 6.43 | 19.72 | 16.51 |
| *WDR4* | 10785 | 7.63 | 22.66 | 20.84 |
| *GPNMB* | 10457 | 0.62 | 1.56 | 1.98 |
| *GEMIN5* | 25929 | 7.99 | 21.78 | 23.94 |
| *CLUH* | 23277 | 14.01 | 44.41 | 35.82 |
| *CGN* | 57530 | 0.33 | 0.94 | 0.95 |
| *STIP1* | 10963 | 145.12 | 390.62 | 445.03 |
| *TBL3* | 10607 | 5.24 | 17.92 | 12.48 |
| *PTPN6* | 5777 | 1.84 | 6.92 | 3.76 |
| *FBXW4P1* | 26226 | 1.22 | 3.64 | 3.5 |
| *SRM* | 6723 | 22.1 | 84.08 | 46.17 |
| *RRP7BP* | 91695 | 9.92 | 30.37 | 28.35 |
| *EBNA1BP2* | 10969 | 43.08 | 122.96 | 132.64 |
| *HSPE1* | 3336 | 104.65 | 277.37 | 347.37 |
| *TUBA4A* | 7277 | 32.85 | 111.34 | 84.79 |
| *PROB1* | 389333 | 1.52 | 5.14 | 3.94 |
| *PDSS1* | 23590 | 5.49 | 16.54 | 16.36 |
| *CCDC86* | 79080 | 22.69 | 73.25 | 62.84 |
| *MIR17HG* | 407975 | 2.36 | 5.79 | 8.41 |
| *RGS20* | 8601 | 1.26 | 4.21 | 3.39 |
| *PRDX1* | 5052 | 130.51 | 380.16 | 408.91 |
| *FLNA* | 2316 | 13.3 | 47.9 | 32.68 |
| *ANKLE1* | 126549 | 8.15 | 23.8 | 25.69 |
| *LDHA* | 3939 | 166.51 | 478.58 | 535.83 |
| *FAM98A* | 25940 | 12.98 | 39.67 | 39.8 |
| *CLDN23* | 137075 | 1.11 | 4.41 | 2.41 |
| *ADAM15* | 8751 | 2.26 | 7.93 | 5.98 |
| *ABCB6* | 10058 | 2.1 | 8.04 | 5.14 |
| *HSP90AB1* | 3326 | 346.45 | 953.09 | 1225.31 |
| *DPF1* | 8193 | 1.14 | 3.65 | 3.57 |
| *SSSCA1* | 10534 | 12.54 | 49.64 | 29.93 |
| *POP1* | 10940 | 3.79 | 8.96 | 15.19 |
| *NOP16* | 51491 | 27.08 | 88.28 | 85.18 |
| *LMNA* | 4000 | 0.96 | 3.95 | 2.2 |
| *SLC29A1* | 2030 | 7.27 | 25.4 | 21.59 |
| *PNP* | 4860 | 9.91 | 32.42 | 31.71 |
| *FABP5* | 2171 | 83.62 | 252.77 | 292.39 |
| *EHD4* | 30844 | 4.95 | 18.58 | 13.99 |
| *NPNT* | 255743 | 1.12 | 3.04 | 4.41 |
| *TUBA1C* | 84790 | 42 | 136.33 | 144.9 |
| *SPHK1* | 8877 | 1.68 | 6.28 | 5.02 |
| *MYO1G* | 64005 | 10.76 | 48.25 | 24.44 |
| *MAP1A* | 4130 | 0.79 | 3.35 | 2.03 |
| *SERINC2* | 347735 | 1.68 | 7.73 | 3.72 |
| *ZNF296* | 162979 | 1.94 | 6.98 | 6.32 |
| *TERT* | 7015 | 1.85 | 7.55 | 5.22 |
| *DLX3* | 1747 | 0.42 | 1.34 | 1.59 |
| *TFRC* | 7037 | 23.26 | 79.68 | 83.12 |
| *EEF1A2* | 1917 | 1.18 | 3.48 | 4.8 |
| *STEAP1B* | 256227 | 1.02 | 2.67 | 4.53 |
| *CDH24* | 64403 | 1.11 | 5.06 | 2.81 |
| *DUSP6* | 1848 | 0.2 | 0.8 | 0.62 |
| *SNORA64* | 26784 | 7.03 | 24.61 | 26.39 |
| *PUS7* | 54517 | 9.5 | 31.79 | 37.2 |
| *AHSA1* | 10598 | 63.73 | 216.19 | 250.73 |
| *MANF* | 7873 | 46.7 | 186.27 | 159.74 |
| *DNAJA1* | 3301 | 100.49 | 320.13 | 427.02 |
| *NDRG4* | 65009 | 0.22 | 0.82 | 0.82 |
| *SLC4A11* | 83959 | 0.51 | 2.12 | 1.71 |
| *NQO1* | 1728 | 2.33 | 11.11 | 6.54 |
| *SLCO4A1* | 28231 | 0.43 | 1.46 | 1.8 |
| *SH2D2A* | 9047 | 0.7 | 2.32 | 3.01 |
| *ACOT7* | 11332 | 9.9 | 40.04 | 35.75 |
| *HYPK* | 25764 | 3.56 | 12.8 | 14.74 |
| *PACSIN3* | 29763 | 1.04 | 4.66 | 3.5 |
| *HIC1* | 3090 | 0.47 | 2.11 | 1.67 |
| *HBEGF* | 1839 | 10.28 | 51.26 | 32.12 |
| *MDK* | 4192 | 5.37 | 32.16 | 13.47 |
| *B4GALNT1* | 2583 | 0.86 | 3.54 | 3.84 |
| *SEPW1* | 6415 | 5.72 | 26.67 | 22.59 |
| *CD151* | 977 | 0.7 | 3.59 | 2.48 |
| *RN7SK* | 125050 | 2.84 | 11.92 | 12.89 |
| *HSP90AA1* | 3320 | 123.18 | 511.74 | 585.84 |
| *MFSD2A* | 84879 | 0.75 | 3.11 | 3.63 |
| *LRFN4* | 78999 | 0.82 | 4.13 | 3.3 |
| *TMEM255A* | 55026 | 0.43 | 2.38 | 1.54 |
| *SNORD86* | 692201 | 41.46 | 209.8 | 170.48 |
| *HPDL* | 84842 | 1.44 | 7.39 | 6.15 |
| *DDN* | 23109 | 1.32 | 6.45 | 6.32 |
| *HSP90B1* | 7184 | 19.12 | 88.21 | 97.72 |
| *HSPH1* | 10808 | 30.06 | 124.94 | 183.07 |
| *TLCD1* | 116238 | 0.56 | 3.21 | 2.53 |
| *HSPA1B* | 3304 | 10.75 | 48.76 | 64.04 |
| *AIF1L* | 83543 | 0.32 | 1.88 | 1.51 |
| *FKBP4* | 2288 | 10.95 | 55.81 | 62.33 |
| *NPTX1* | 4884 | 0.16 | 1 | 0.74 |
| *CALR* | 811 | 116.09 | 753.75 | 562.97 |
| *SDF2L1* | 23753 | 5.37 | 42.77 | 18.28 |
| *NRARP* | 441478 | 0.23 | 1.48 | 1.17 |
| *DUSP2* | 1844 | 0.41 | 3.1 | 2.05 |
| *SH2D5* | 400745 | 0.27 | 1.91 | 1.56 |
| *LINC01559* | 283422 | 0.31 | 2.06 | 1.93 |
| *SNORD79* | 26770 | 6.6 | 36.67 | 49.26 |
| *HSPA5* | 3309 | 39.58 | 258.14 | 260.21 |
| *AHNAK* | 79026 | 0.16 | 0.43 | 2.04 |
| *HSPA8* | 3312 | 134.91 | 1070.65 | 1157.07 |
| *ZHX1-C8orf76* | 1.01E+08 | 0.44 | 1.58 | 6.4 |
| *HSPA1A* | 3303 | 0.53 | 4.17 | 6.23 |
| *ACTN1* | 87 | 0.11 | 0.99 | 1.17 |
| *SLC17A9* | 63910 | 0.34 | 4.14 | 2.79 |
| *LOC100507091* | 1.01E+08 | 0.05 | 0.53 | 0.53 |
| *COL8A2* | 1296 | 0.04 | 0.47 | 0.44 |
| *HSD11B2* | 3291 | 0.1 | 0.91 | 1.4 |
| *LOC389247* | 389247 | 0.14 | 1.72 | 1.59 |
| *CAMKV* | 79012 | 0.03 | 0.51 | 0.38 |
| *LOC101927438* | 1.02E+08 | 0.05 | 0.84 | 0.78 |
| *NUTM2A* | 728118 | 0.01 | 0.29 | 0.32 |
| *TBC1D3* | 729873 | 0.01 | 0.68 | 0.58 |
| *FAM35DP* | 439965 | 0.01 | 0.84 | 0.76 |
| *POC1B-GALNT4* | 1.01E+08 | 0.01 | 0.69 | 0.96 |
| *KLHL23* | 151230 | 0.01 | 0.48 | 1.52 |
| *LOC541473* | 541473 | 0.01 | 1.22 | 1.39 |
| *EGFL8* | 80864 | 0.01 | 1.84 | 1.73 |
| *BORCS7-ASMT* | 1.01E+08 | 0.01 | 2.52 | 1.28 |
| *SERF1A* | 8293 | 0.01 | 3.04 | 0.9 |
| *TNFAIP8L2-SCNM1* | 1.01E+08 | 0.01 | 3.95 | 0.68 |
| *LOC388436* | 388436 | 0.01 | 0.99 | 4.04 |
| *HSPE1-MOB4* | 1.01E+08 | 0.01 | 3.88 | 4.68 |
| *SGK3* | 23678 | 0.01 | 6.39 | 3.67 |
| *SNORD19B* | 1E+08 | 0.01 | 25.49 | 18.56 |

**Supplemental References**

1. Compagno M, Lim WK, Grunn A, Nandula SV, Brahmachary M, Shen Q, et al. Mutations of multiple genes cause deregulation of NF-kappaB in diffuse large B-cell lymphoma*. Natu*re 2009**; 4**59: 717-21.
