# Supplementary figures and images for: FBXL10 contributes to the development of diffuse large B-cell lymphoma by epigenetically enhancing ERK1/2 signaling pathway
Source: Cell Death Dis. 2018 Jan 19;9(2):46. doi: 10.1038/s41419-017-0066-8 (PMC5833345; doi:10.1038/s41419-017-0066-8)

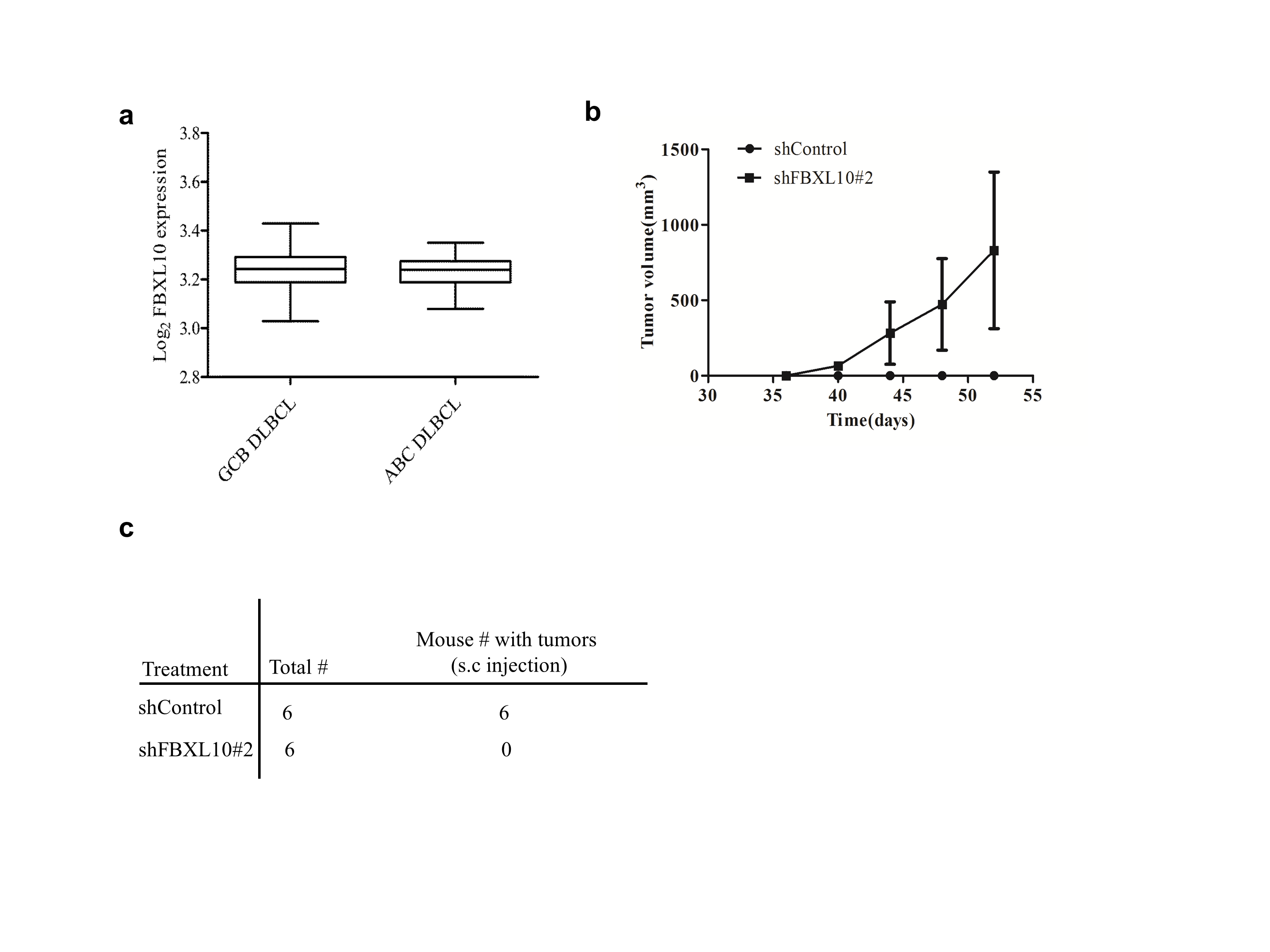

Supplement: Supplementary file 2 — Figure S1 [file 41419_2017_66_MOESM2_ESM.tif]

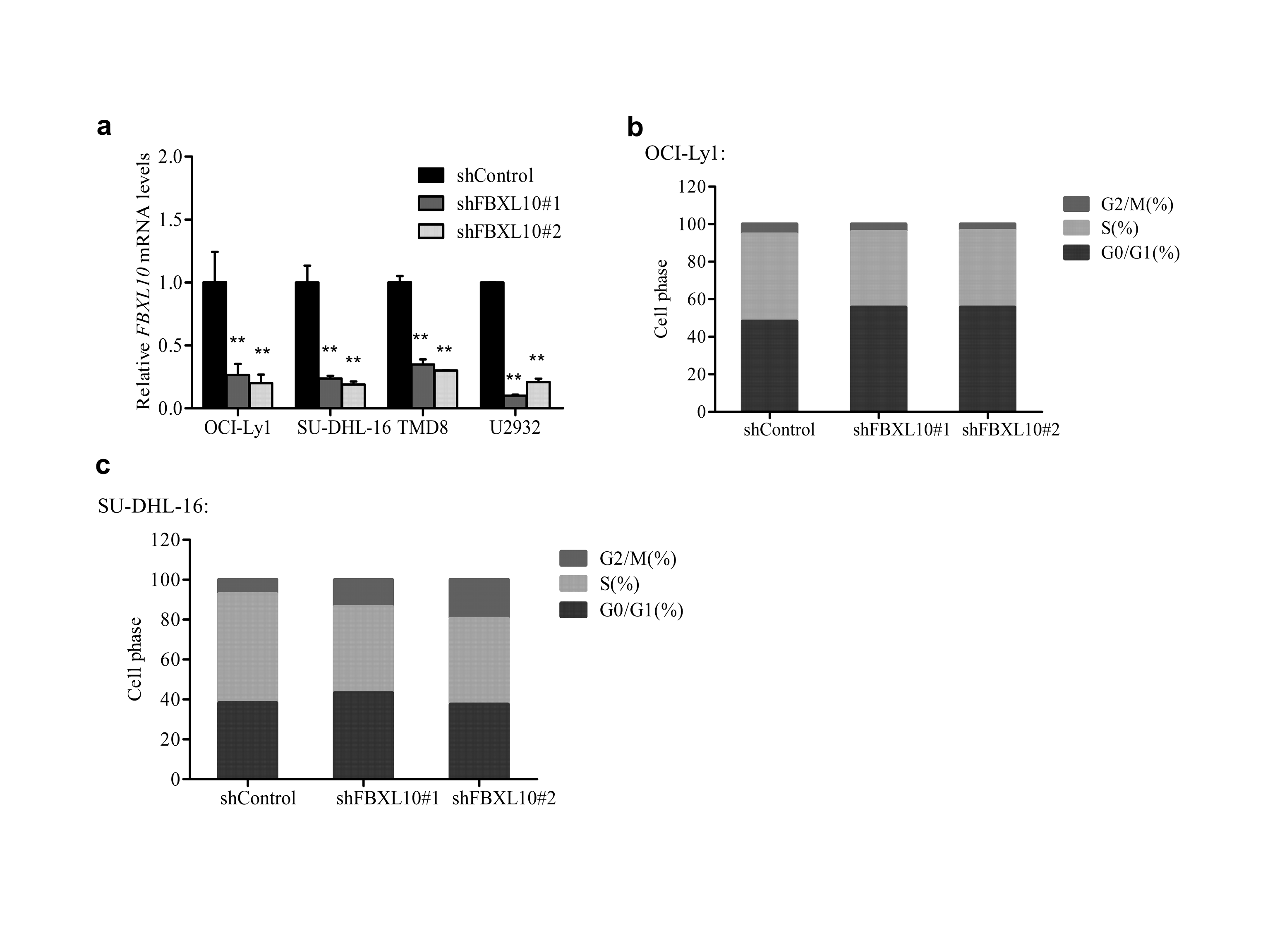

Supplement: Supplementary file 3 — Figure S2 [file 41419_2017_66_MOESM3_ESM.tif]

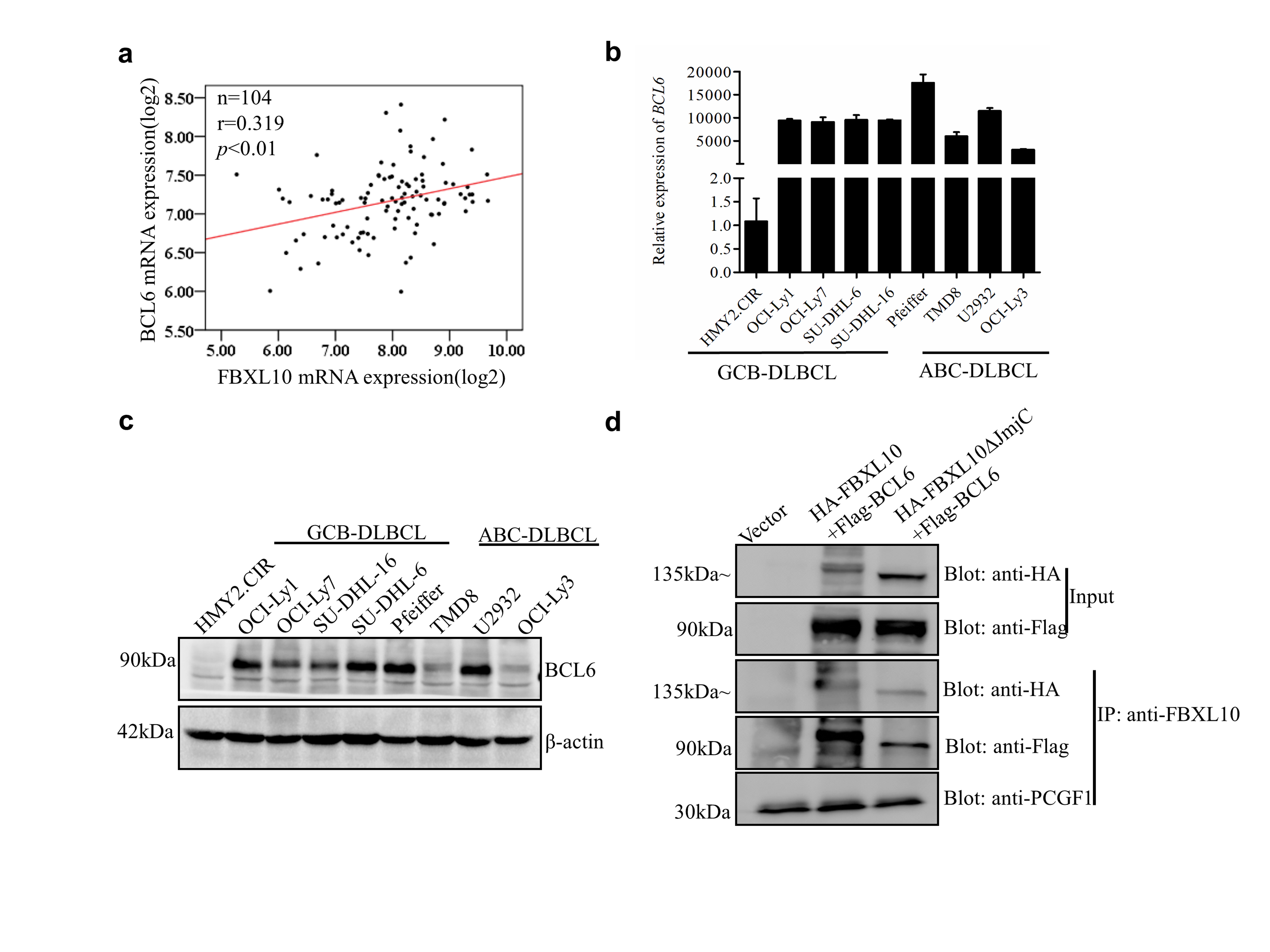

Supplement: Supplementary file 4 — Figure S3 [file 41419_2017_66_MOESM4_ESM.tif]

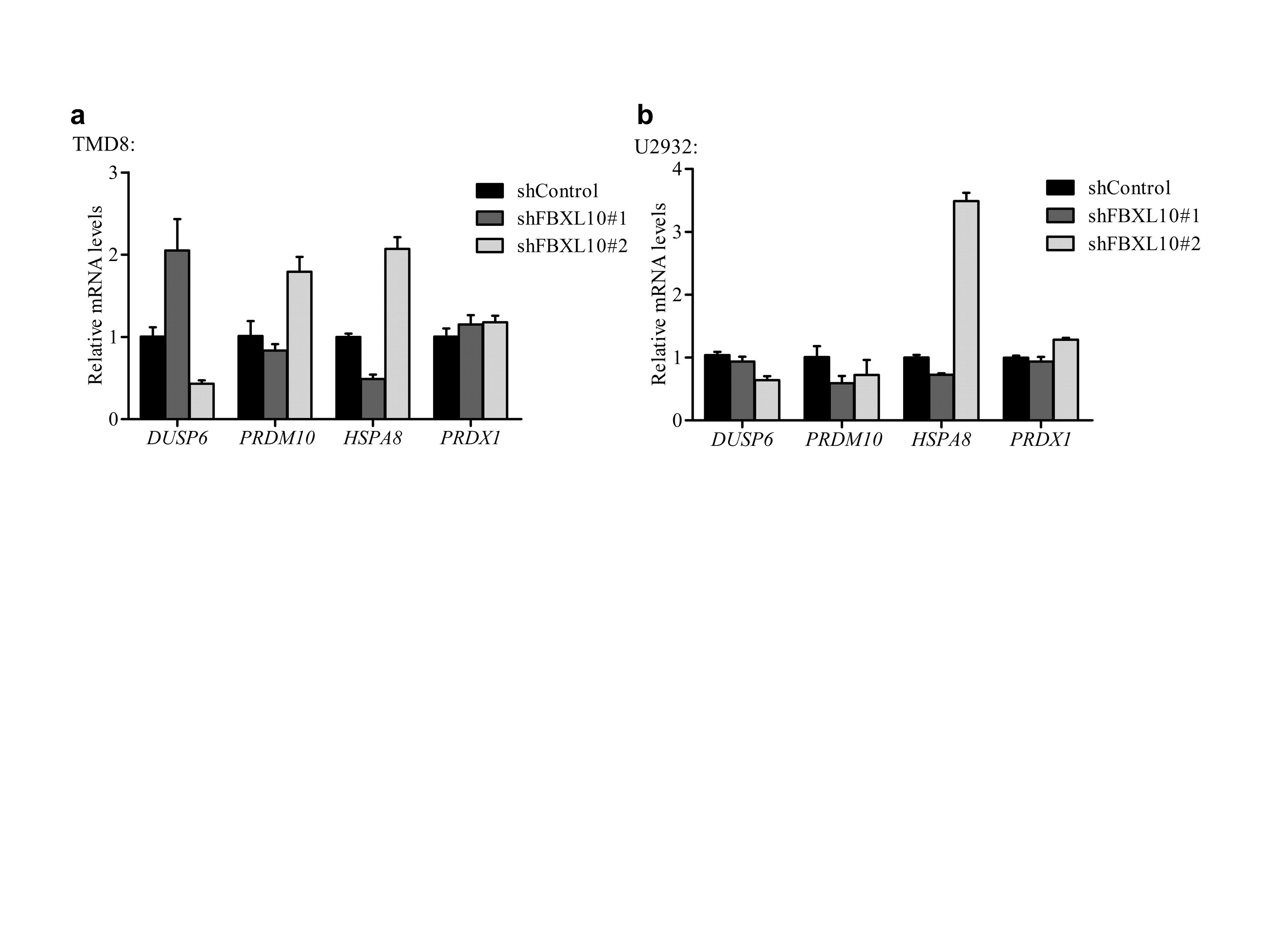

Supplement: Supplementary file 5 — Figure S4 [file 41419_2017_66_MOESM5_ESM.tif]
